# Use of SGLT2 Inhibitors vs GLP-1 RAs and Anemia in Patients With Diabetes and CKD

**DOI:** 10.1001/jamanetworkopen.2024.0946

**Published:** 2024-03-04

**Authors:** Jia-Chian Hu, Shih-Chieh Shao, Daniel Hsiang-Te Tsai, Albert Tzu-Ming Chuang, Kuan-Hung Liu, Edward Chia-Cheng Lai

**Affiliations:** 1 School of Pharmacy, Institute of Clinical Pharmacy and Pharmaceutical Sciences, College of Medicine, National Cheng Kung University, Tainan, Taiwan; 2 Department of Pharmacy, Keelung Chang Gung Memorial Hospital, Keelung, Taiwan; 3 Institute of Clinical Medicine, College of Medicine, National Cheng Kung University, Tainan, Taiwan.; 4 Division of Nephrology, Department of Internal Medicine, National Cheng Kung University Hospital, College of Medicine, National Cheng Kung University, Tainan, Taiwan

## Abstract

**Importance:**

Sodium-glucose cotransporter 2 (SGLT2) inhibitors are associated with lower anemia risk, based on findings from post hoc analyses of the CREDENCE and DAPA-CKD trials; however, the effectiveness of SGLT2 inhibitors in a more generalizable type 2 diabetes (T2D) and chronic kidney disease (CKD) population, with active comparisons pertinent to current practice, is unknown.

**Objective:**

To evaluate and compare anemia incidence between SGLT2 inhibitors and glucagon-like peptide-1 receptor agonists (GLP-1 RAs) among patients with T2D and CKD stages 1 to 3.

**Design, Setting, and Participants:**

This retrospective cohort study used target trial emulation of an expanded CREDENCE and DAPA-CKD study framework. The study was conducted among adults with T2D and CKD initiating SGLT2 inhibitors or GLP-1 RAs between January 1, 2016, and December 31, 2021, with follow-up until December 31, 2022. The study was conducted at the Chang Gung Medical Foundation, the largest multi-institutional hospital system in Taiwan.

**Exposures:**

Initiation of SGLT2 inhibitors or GLP-1 RAs.

**Main Outcomes and Measures:**

The primary outcome was a composite of anemia outcomes, including anemia event occurrence (hemoglobin level <12-13 g/dL or *International Statistical Classification of Diseases, Tenth Revision, Clinical Modification *diagnosis codes) or anemia treatment initiation. Changes in hematological parameters, including hemoglobin level, hematocrit level, and red blood cell count, were evaluated during the follow-up period for as long as 3 years.

**Results:**

The cohort included a total of 13 799 patients with T2D and CKD, initiating SGLT2 inhibitors (12 331 patients; mean [SD] age, 62.4 [12.3] years; 7548 [61.2%] male) or GLP-1 RAs (1468 patients; mean [SD] age, 61.5 [13.3] years; 900 [61.3%] male). After the median follow-up period of 2.5 years, patients receiving SGLT2 inhibitors had lower incidence of composite anemia outcomes (hazard ratio [HR], 0.81; 95% CI, 0.73-0.90) compared with those receiving GLP-1 RAs. SGLT2 inhibitors were associated with a lower incidence of anemia events (HR, 0.79; 95% CI, 0.71-0.87) but not with a lower rate of anemia treatment initiation (HR, 0.99; 95% CI, 0.83-1.19). Changes in hematological parameters for SGLT2 inhibitors and GLP-1 RAs throughout the 3-year follow-up period supported the primary analyses.

**Conclusions and Relevance:**

In this multi-institutional cohort study with target trial emulation, SGLT2 inhibitors were associated with a decreased risk of composite anemia outcomes, especially anemia event occurrences. SGLT2 inhibitors may be considered as an adjunct therapy to reduce anemia incidence in patients with T2D and CKD.

## Key Points

**Question:**

Is the use of sodium-glucose cotransporter 2 (SGLT2) inhibitors associated with reduced risk of anemia in patients with type 2 diabetes (T2D) and chronic kidney disease (CKD) stages 1 to 3?

**Findings:**

In this cohort study with 13 799 participants, initiation of SGLT2 inhibitors was associated with a 19% decrease in incident anemia risk compared with initiation of glucagon-like peptide-1 receptor agonists. Changes in hemoglobin, hematocrit, and red blood cell counts during the follow-up period supported this observation.

**Meaning:**

The study findings suggest potential benefits for anemia incidence after SGLT2 inhibitor initiation in patients with T2D and CKD.

## Teaser

This cohort study compares anemia incidence between sodium-glucose cotransporter 2 (SGLT2) inhibitors and glucagon-like peptide-1 receptor agonists (GLP-1 RAs) among patients with type 2 diabetes and chronic kidney disease (CKD) stages 1 to 3 in Taiwan.

## Introduction

Anemia, defined as having hemoglobin levels less than 13 g/dL in men and less than 12 g/dL in women (to convert hemoglobin to grams per liter, multiply by 10), occurs in 20% to 30% of patients with type 2 diabetes (T2D) and chronic kidney disease (CKD)^1,2^ and is associated with higher mortality and health-related poor quality of life.^3,4,5,6,7^ Currently, clinical practice guidelines lack specific recommendations for adjuvant therapy to prevent the occurrence of anemia.^8^ To mitigate anemia risk, patients with T2D and CKD should achieve their glycemic goals and maintain kidney functions by using appropriate glucose-lowering drugs, including sodium-glucose cotransporter 2 (SGLT2) inhibitors or glucagon-like peptide-1 receptor agonists (GLP-1 RAs) with proven additional kidney benefits.^9,10^

A meta-analysis of large clinical trials has shown that compared with placebo, SGLT2 inhibitors are associated with a 38% reduced risk of kidney outcomes,^11^ probably because SGLT2 inhibitors decrease diabetic glomerular hyperfiltration due to hyperglycemia.^12^ In addition to glucose-lowering and beneficial kidney effects, SGLT2 inhibitors have been shown to increase hemoglobin levels in patients with T2D and CKD.^13^ Recently, 2 post hoc analyses of the CREDENCE (Canagliflozin and Renal Events in Diabetes With Established Nephropathy Clinical Evaluation) and DAPA-CKD (Dapagliflozin and Prevention of Adverse Outcomes in Chronic Kidney Disease) trials have suggested additional benefits regarding anemia-related outcomes from canagliflozin and dapagliflozin treatment.^14,15^ Although the exact mechanism behind these findings remains underinvestigated, SGLT2 inhibitors appear linked to increases in erythropoietin (EPO) production, contributing to increased oxygen delivery to the kidney and decreased kidney hypoxia.^16,17^

Despite these promising post hoc analysis findings,^14,15^ limitations in the design of the CREDENCE and DAPA-CKD trials may have reduced their relevance for clinical practice. The trials’ strict eligibility requirements (severe proteinuria) may have lowered the proportion of trial participants who accurately reflect patients receiving SGLT2 inhibitors in clinical practice.^18^ A previous study^19^ also reported better kidney function in terms of estimated glomerular filtration rate (eGFR) values in patients newly initiating SGLT2 inhibitors under routine care (mean eGFR, 85-92 mL/min/1.73 m^2^) compared with the participants in the CREDENCE (mean eGFR, 56 mL/min/1.73 m^2^) and DAPA-CKD trials (mean eGFR, 43 mL/min/1.73 m^2^).^14,15^ Finally, these clinical trials compared the efficacy of SGLT2 inhibitors with a placebo group, leaving uncertainty about whether anemia effects were independent of glycemic controls and kidney function improvements after SGLT2 inhibitor treatment. Hence, further studies with more generalizable populations and active comparisons pertinent to current practice in patients with T2D and CKD are warranted.

In this, Taiwan’s largest multi-institutional cohort study emulating the CREDENCE and DAPA-CKD trials, we investigated anemia incidence associated with initiation of SGLT2 inhibitors, focusing on patients with T2D and CKD stages 1 to 3. We compared SGLT2 inhibitors with GLP-1 RAs, which do not exhibit anti-anemic effects but are similarly positioned within the T2D treatment algorithm.^20,21^

## Methods

### Data Source

This study analyzed the Chang Gung Research Database (CGRD), which collects deidentified electronic medical records (EMRs) from 7 Chang Gung Memorial Hospitals, Taiwan’s largest health care system covering more 10 000 beds and 8.7 million annual outpatient visits.^22,23^ Diagnoses recorded in the CGRD used the *International Classification of Diseases, Ninth Revision, Clinical Modification* (*ICD-9-CM*) codes prior to 2016 and *International Classification of Diseases, 10th Revision, Clinical Modification* (*ICD-10-CM*) codes thereafter.^22^ Records of medications in the CGRD are based on prescription claims. The accuracy of diagnostic codes in the CGRD has been extensively validated.^24,25,26^

The CGRD is an important data source that has provided clinical evidence for previous pharmacoepidemiologic studies.^27,28,29^ Notably, the CGRD includes laboratory information (eg, glycemic and kidney function data), allowing the evaluation of T2D disease severity, enabling more precise results in comparative drug effectiveness research.^30,31,32^ We followed the Reporting of Studies Conducted Using Observational Routinely Collected Health Data Statements for Pharmacoepidemiology (RECORD-PE) guidelines in reporting our results, and our study protocol was approved by the institutional review board of Chang Gung Medical Foundation.^33^ Informed consent was waived due to the retrospective collection of anonymized data.

### Study Design

To strengthen the robustness of this observational study, we followed the target trial emulation design framework, modified from the CREDENCE and DAPA-CKD studies (eFigure 1 and eTable 1 in Supplement 1) and adopted a new-user design to compare drug outcomes between SGLT2 inhibitors and an active comparator. While dipeptidyl peptidase-4 (DPP-4) inhibitors, including saxagliptin and linagliptin, have proven kidney benefits for patients with T2D and CKD,^34,35^ we chose GLP-1 RAs as the active comparator in this study because (1) they share similar pleiotropic effects with SGLT2 inhibitors, such as cardio-kidney and metabolic impacts^36,37,38^; (2) they have similar treatment positions to SGLT2 inhibitors, according to the current practice guidelines^39,40^; and (3) they are frequently selected as the comparison group in comparative effectiveness and safety studies of SGLT2 inhibitors.^41,42,43^ This design choice enabled us to evaluate whether SGLT2 inhibitors were associated with a reduced risk of anemia in adults with T2D and CKD stages 1 to 3.

### Study Cohort

We included patients with T2D aged older than 18 years with hemoglobin A_1c_ (HbA_1c_) levels of 6.5% or greater (to convert to proportion of total hemoglobin, multiply by 0.01) and CKD stages 1 to 3, newly receiving SGLT2 inhibitors or GLP-1 RAs between January 1, 2016, and December 31, 2021. The index date was defined as the first prescription date for SGLT2 inhibitors or GLP-1 RAs. To include more generalizable T2D and CKD populations, we included patients with CKD stages 1 to 3 if their (1) eGFR levels were between 30 and 60 mL/min/1.73 m^2^ or (2) their eGFR levels were greater than 60 mL/min/1.73 m^2^ and their urine albumin-creatinine ratio (UACR) was greater than 30 mg/g, using the most recent laboratory information recorded within 1 year before the index date.^44^ Specifically, we did not include patients with stable doses of angiotensin receptor blocker or angiotensin-converting enzyme inhibitor treatment before the index date, one of the major inclusion criteria of the CREDENCE and DAPA-CKD trials, because underuse of these medications in patients with T2D in clinical practice has been reported.^45^ A comparison of eligibility criteria between the CREDENCE and DAPA-CKD trials and this study is presented in eTable 2 in Supplement 1. We also adopted the major exclusion criteria (eg, history of diabetic ketoacidosis or type 1 diabetes, nephrotic syndrome, kidney transplant, cancer, HIV) of the CREDENCE and DAPA-CKD trials.^46,47^ Finally, to identify incident cases, we excluded patients with anemia events, based on the clinical diagnosis, laboratory data, and treatments 12 weeks prior to the index date. Details and definitions of these exclusion criteria are listed in eTables 3 and 4 in Supplement 1.

### Propensity Scores

We used propensity scores with fine stratification weights to generate similar probability-of-treatment assignment between the SGLT2 inhibitor and GLP-1 RA groups.^48^ Compared with propensity score matching or traditional weighting methods, this approach retained the maximum number of study patients and provided better confounding controls while generating fewer extreme weights.^48,49,50^ We considered a wide range of potential confounders based on the target trials and other previous literature to calculate propensity scores.^1,14,15,51^ These covariates used for propensity score estimations included demographic characteristics, laboratory information, comorbidities (identified at least once using *ICD-9-CM* or *ICD-10-CM* codes), and comedications listed in Table 1, all derived from EMR in the outpatient setting. We present the details of baseline comorbidities and comedications in eTables 5 and 6 in Supplement 1. In clinical practice, patients with T2D may not routinely receive UACR, body mass index (BMI; calculated as weight in kilograms divided by height in meters squared), hemoglobin, and hematocrit tests. For example, based on Taiwan National Health Insurance claims data, it has been estimated that only approximately 40% of patients with T2D receive UACR tests.^52^ To address the issue of missing laboratory data, we categorized the absence of these variables as no measurement. The propensity score was then generated using multivariable logistic regression models that utilized the covariates mentioned previously.^50^ After creating the propensity score distributions, we trimmed patients from nonoverlapping regions and created 50 equal strata based on the propensity score distribution of the patients who received SGLT2 inhibitors.^50^ We calculated the average treatment effect weights for patients treated with SGLT2 inhibitors or GLP-1 RAs.^48^ This calculation involved dividing the proportion of total patients in each stratum of the overall population by the proportion of patients in the same stratum who were receiving SGLT2 inhibitors or GLP-1 RAs out of the total number of patients treated with these drugs.^48,49^

**Table 1.** Patient Characteristics Before and After Applying Propensity-Score Fine Stratification Weights

Characteristic	Original cohort			Study cohort after fine stratification weighting		
	Patients, No. (%)		SMD^a^	Patients, No. (%)		SMD^a^
	SGLT2 inhibitors (n = 12 463)	GLP-1 RAs (n = 1479)		SGLT2 inhibitors (n = 12 331)	GLP-1 RAs (n = 1468)	
Age, y						
Mean (SD)	62.7 (12.2)	61.7 (13.7)	0.07	62.4 (12.3)	61.5 (13.3)	0.08
18-40	537 (4.3)	97 (6.6)	−0.10	568 (4.6)	87 (5.9)	−0.06
40-64	6032 (48.4)	696 (47.1)	0.03	6000 (48.7)	708 (48.2)	0.01
>65	5894 (47.3)	686 (46.4)	0.02	5763 (46.7)	673 (45.9)	0.02
Sex						
Male	7810 (62.7)	771 (52.1)	0.21	7548 (61.2)	900 (61.3)	0.00
Female	4653 (37.3)	708 (47.9)		4783 (38.8)	568 (38.7)	
HbA_1c_, %						
Median (IQR)	8.3 (7.4 to 9.6)	9.4 (8.4 to 10.5)	−0.52	8.4 (7.5 to 9.7)	8.6 (7.6 to 9.7)	0.00
6.5-8.0	4985 (40.0)	226 (15.3)	0.58	4545 (36.9)	555 (37.8)	−0.02
8.0-9.5	4138 (33.2)	540 (36.5)	−0.07	4172 (33.8)	489 (33.3)	0.01
>9.5	3340 (26.8)	713 (48.2)	−0.45	3615 (29.3)	425 (28.9)	0.01
eGFR, mL/min/1.73 m^2^						
Median (IQR)	69.0 (54.0 to 90.4)	62.0 (45.7 to 90.0)	0.11	69.0 (53.0 to 90.7)	69.3 (52.1 to 90.0)	0.00
≥90	3225 (25.9)	373 (25.2)	0.02	3208 (26.0)	368 (25.1)	0.02
60-89	4454 (35.7)	394 (26.6)	0.20	4289 (34.8)	556 (37.9)	−0.06
45-59	3515 (28.2)	359 (24.3)	0.09	3407 (27.6)	367 (25.0)	0.06
30-44	1269 (10.2)	353 (23.9)	−0.37	1427 (11.6)	177 (12.1)	−0.02
UACR, mg/g						
Median (IQR)	98.6 (37.9 to 305.1)	120.1 (46.0 to 450.9)	−0.11	101.0 (38.5 to 316.5)	101.0 (40.0 to 352.8)	0.02
<30	1243 (10.0)	131 (8.9)	0.04	1212 (9.8)	128 (8.7)	0.04
30-300	7252 (58.2)	803 (54.3)	0.08	7137 (57.9)	851 (58.0)	0.00
≥300	3026 (24.3)	456 (30.8)	−0.15	3092 (25.1)	389 (26.5)	−0.03
No measurement	942 (7.6)	89 (6.0)	0.06	890 (7.2)	101 (6.9)	0.01
BMI						
Median (IQR)	27.5 (24.9 to 30.7)	28.3 (25.3 to 31.8)	−0.18	27.6 (24.9 to 30.8)	28.0 (25.2 to 31.6)	−0.14
<24	1615 (13.0)	176 (11.9)	0.03	1578 (12.8)	171 (11.6)	0.04
>24-30	4870 (39.1)	588 (39.8)	−0.01	4840 (39.3)	518 (35.3)	0.08
30	2711 (21.8)	441 (29.8)	−0.19	2817 (22.8)	367 (25.0)	−0.05
No measurement	3267 (26.2)	274 (18.5)	0.19	3096 (25.1)	412 (28.0)	−0.07
Hemoglobin, g/dL						
Median (IQR)	13.8 (12.6 to 15.0)	13.6 (12.3 to 14.7)	0.12	13.8 (12.6 to 15.0)	13.9 (12.6 to 14.8)	0.03
Female <13 g/dL or male <14 g/dL	3453 (27.7)	416 (28.1)	−0.01	3408 (27.6)	422 (28.8)	−0.03
Female >13 g/dL or male >14 g/dL	4302 (34.5)	467 (31.6)	0.06	4217 (34.2)	491 (33.4)	0.02
No measurement	4708 (37.8)	596 (40.3)	−0.05	4706 (38.2)	555 (37.8)	0.01
Hematocrit, %						
Median (IQR)	41.1 (38.0 to 44.4)	40.2 (36.8 to 43.3)	0.20	41.0 (37.9 to 44.3)	41.5 (38.1 to 44.5)	−0.01
<40	2244 (18.0)	358 (24.2)	−0.15	2291 (18.6)	245 (16.7)	0.05
≥40	3362 (27.0)	387 (26.2)	0.02	3302 (26.8)	414 (28.2)	−0.03
No measurement	6857 (55.0)	734 (49.6)	0.11	6738 (54.6)	809 (55.1)	−0.01
Heart failure	851 (6.8)	64 (4.3)	0.11	747 (6.1)	82 (5.6)	0.02
Ischemic heart disease	1451 (11.6)	116 (7.8)	0.13	1330 (10.8)	129 (8.8)	0.07
Peripheral arterial disease	234 (1.9)	38 (2.6)	−0.05	240 (1.9)	28 (1.9)	0.00
Ischemic stroke	784 (6.3)	80 (5.4)	0.04	756 (6.1)	126 (8.6)	−0.09
Atrial fibrillation	489 (3.9)	45 (3.0)	0.05	460 (3.7)	45 (3.1)	0.04
Hypertension	8679 (69.6)	1043 (70.5)	−0.02	8587 (69.6)	970 (66.1)	0.08
Dyslipidemia	8398 (67.4)	1040 (70.3)	−0.06	8359 (67.8)	951 (64.8)	0.06
Autoinflammatory diseases	41 (0.3)	5 (0.3)	0.00	42 (0.3)	8 (0.5)	−0.03
Respiratory disease	696 (5.6)	87 (5.9)	−0.01	681 (5.5)	71 (4.8)	0.03
Thyroid gland disorder	277 (2.2)	55 (3.7)	−0.09	299 (2.4)	34 (2.3)	0.01
Diabetes medications						
Insulin	2164 (17.4)	750 (50.7)	−0.75	2595 (21.0)	317 (21.6)	−0.01
Metformin	8687 (69.7)	929 (62.8)	0.15	8500 (68.9)	959 (65.4)	0.08
Sulfonylurea	6166 (49.5)	810 (54.8)	−0.11	6204 (50.3)	694 (47.3)	0.06
α-Glucosidase inhibitor	1657 (13.3)	282 (19.1)	−0.16	1717 (13.9)	204 (13.9)	0.00
Thiazolidinedione	1827 (14.7)	211 (14.3)	0.01	1813 (14.7)	228 (15.5)	−0.02
DPP-4 inhibitor	7169 (57.5)	997 (67.4)	−0.21	7239 (58.7)	822 (56.0)	0.05
Meglitinide	268 (2.2)	78 (5.3)	−0.17	296 (2.4)	30 (2.1)	0.02
Vitamin B_12_ or folic acid	657 (5.3)	126 (8.5)	−0.13	691 (5.6)	65 (4.4)	0.05
ACE inhibitors or ARBs	9548 (76.6)	1139 (77.0)	−0.01	9437 (76.5)	1093 (74.5)	0.05
Diuretics	3102 (24.9)	405 (27.4)	−0.06	3064 (24.8)	368 (25.1)	−0.01
β-Blockers	5533 (44.4)	612 (41.4)	0.06	5384 (43.7)	598 (40.7)	0.06
Calcium channel blockers	4696 (37.7)	605 (40.9)	−0.07	4684 (38.0)	514 (35.0)	0.06
Lipid-modifying agents	10 351 (83.1)	1234 (83.4)	−0.01	10 231 (83.0)	1208 (82.3)	0.02
Anticoagulants or antiplatelets	5782 (46.4)	676 (45.7)	0.01	5671 (46.0)	713 (48.6)	−0.05
NSAIDs	5749 (46.1)	743 (50.2)	−0.08	5764 (46.7)	702 (47.8)	−0.02
Systemic glucocorticoids	2630 (21.1)	373 (25.2)	−0.10	2656 (21.5)	318 (21.7)	0.00
PPIs or H2 blockers	4814 (38.6)	588 (39.8)	−0.02	4750 (38.5)	544 (37.1)	0.03
Antiseizure medications	1464 (11.7)	219 (14.8)	−0.09	1491 (12.1)	181 (12.3)	−0.01
Methotrexate	47 (0.4)	5 (0.3)	0.01	46 (0.4)	4 (0.3)	0.02

Abbreviations: ACE, angiotensin-converting enzyme; ARBs, angiotensin receptor blockers; BMI, body mass index (calculated as weight in kilograms divided by height in meters squared); DPP-4, dipeptidyl peptidase-4; eGFR, estimated glomerular filtration rate; GLP-1 RAs, glucagon-like peptide-1 receptor agonists; HbA_1c_, hemoglobin A_1c_; H2, histamine 2 receptor; NSAIDs, nonsteroidal anti-inflammatory drugs; PPIs, proton pump inhibitors; SGLT2, sodium-glucose cotransporter 2; SMD, standardized mean difference; UACR, urine albumin-to-creatinine ratio.

SI conversion factors: To convert HbA_1c_ to proportion of total hemoglobin, multiply by 0.01; hematocrit to proportion of 1.0, multiply by 0.01; hemoglobin to grams per liter, multiply by 10.

^a^ An SMD between −0.1 and 0.1 indicates a negligible statistical difference between the 2 treatment groups.

### Definitions of Drug Exposure and Comparator

We included empagliflozin, dapagliflozin, canagliflozin, and ertugliflozin as the exposure group (SGLT2 inhibitors) and lixisenatide, liraglutide, dulaglutide, and semaglutide as the comparison group (GLP-1 RAs), since these were the drugs available for intensified T2D management during the study period in Taiwan. The Anatomical Therapeutic Chemical codes to identify SGLT2 inhibitors and GLP-1 RAs are listed in eTable 7 in Supplement 1.

### Main Outcome and Measure

We followed the CREDENCE and DAPA-CKD trials to define the primary incident composite anemia outcomes,^14,15^ including (1) incident anemia events (hemoglobin levels: females, <12 g/dL; males; <13 g/dL; or clinical diagnosis of anemia using *ICD-10-CM* diagnosis codes) and (2) initiation of anemia treatments (oral or parenteral iron preparations, erythropoiesis-stimulating agents, or red blood cell transfusion). Detailed outcome definitions are listed in eTable 8 in Supplement 1. Secondary outcomes included the individual components of the primary outcome.

### Changes in Hematological Parameters, Glycemic Control, and Kidney Function

To support the primary outcome analyses, we investigated changes in hemoglobin levels, hematocrit levels, and red blood cell counts in the same propensity-score weighted cohort during the follow-up period. We also obtained the HbA_1c_, eGFR, and UACR values after 1-, 2- and 3-year periods of SGLT2 inhibitor or GLP-1 RA use, based on complete data analyses, seeking to determine possible mechanisms behind the anemia outcomes. Where multiple measurements were available within 1 follow-up period, we only included those closest to the 1-, 2- or 3-year point.

### Follow-Up

Patients were followed up from initiation of SGLT2 inhibitor or GLP-1 RA use to the occurrence of primary outcome or death, or the last clinical visit to a study hospital before the end of the database, whichever came first. The database ended December 31, 2022.

### Statistical Analysis

We used the mean with SD and number with percentage to summarize the baseline characteristics of continuous and categorical variables, respectively. We alternatively used median with IQR for continuous variables where appropriate. To assess differences in baseline characteristics and differences in the changes in hematological parameters, glycemic control, and kidney function between the SGLT2 inhibitor and GLP-1 RA groups during the study follow-up, we used standardized mean differences (SMDs), whereby an SMD between −0.1 and 0.1 indicated a negligible difference between the 2 treatment groups.^53^ Anemia incidence rates (IRs) were presented as number of events per 100 person-years. We used Cox proportional hazards models to estimate hazard ratios (HRs) with 95% CIs of incident anemia outcomes for the 2 groups. We considered a 2-sided *P* < .05 to denote statistical significance. All analyses were conducted with SAS software version 9.4 (SAS Institute Inc).

#### Subgroup Analysis

To compare the risk of composite anemia outcomes between SGLT2 inhibitors and GLP-1 RAs across different patient groups, we conducted subgroup analyses stratified by sex, age (<65 or ≥65 years), HbA_1c_ levels (<8% or ≥8%), eGFR levels (eGFR ≥30 mL/min/1.73 m^2^ to <60 mL/min/1.73 m^2^, ≥60 mL/min/1.73 m^2^ to <90 mL/min/1.73 m^2^, or ≥90 mL/min/1.73 m^2^) and SGLT2 inhibitors (empagliflozin, dapagliflozin, or canagliflozin) by redeveloping a similar propensity score with fine stratification approach for each subgroup analysis. We did not individually analyze ertugliflozin because it was not approved for T2D management in Taiwan until 2021, and case numbers are probably limited.

#### Sensitivity Analysis

To examine the robustness of the primary analysis results, we conducted 3 sensitivity analyses. First, we applied on-treatment analysis to evaluate the association of nonadherence or drug switching after the index date with the study results. In this analysis, we censored patients exceeding 90 days without an index drug prescription refill, patients switching from the initial index drug class to another index drug class, and patients adding another index drug class during follow-up. Second, we excluded patients newly receiving lixisenatide from the comparison group, since clinical features of this drug (eg, short-acting pharmacological effects and lack of kidney and cardiovascular benefits, according to a randomized clinical trial^54^) may differ from other GLP-1 RAs. Third, we extended the period of exclusion due to anemia history from 12 weeks to 1 year prior to the index date to eliminate the effects of remote anemia events.

## Results

### Baseline Characteristics

**Figure 1.**
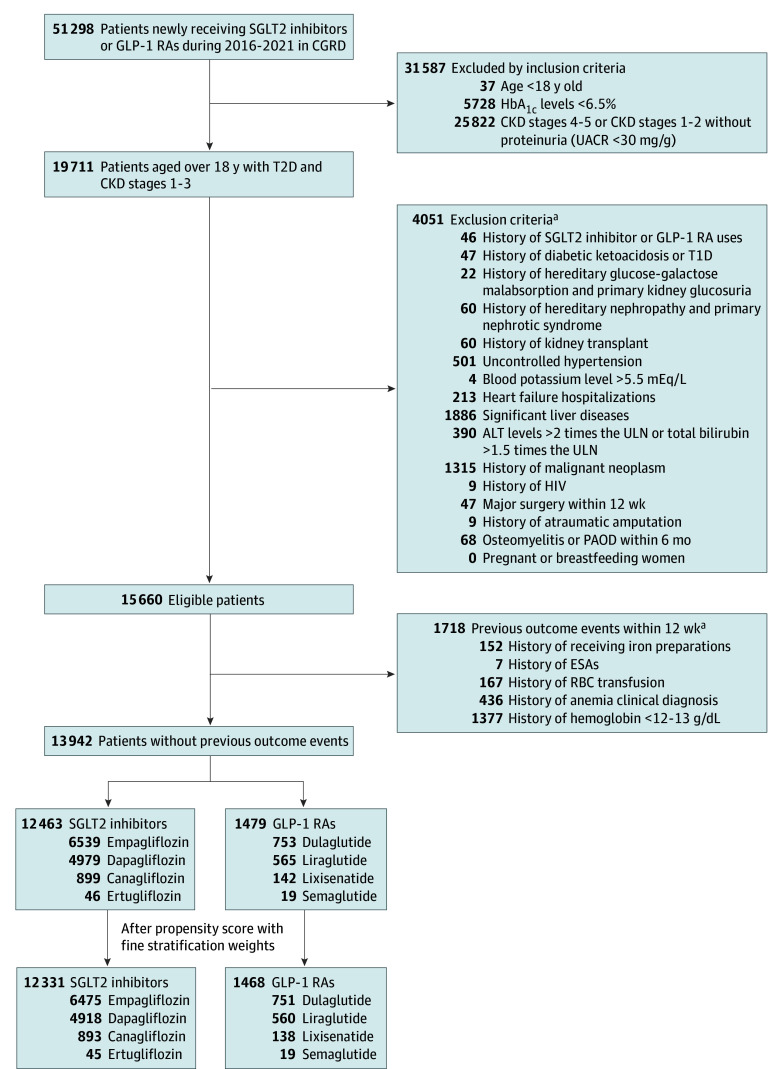
Patient Selection Flowchart To convert hemoglobin to grams per liter, multiply by 10; hemoglobin A_1c_ (HbA_1c_) to proportion of total hemoglobin, multiply by 0.01; potassium to millimoles per liter, multiply by 1. ALT indicates alanine transaminase; CGRD, Chang Gung Research Database; CKD, chronic kidney disease; ESAs, erythropoiesis-stimulating agents; GLP-1 RAs, glucagon-like peptide-1 receptor agonists; PAOD, peripheral arterial occlusion disease; RBC, red blood cell; SGLT2, sodium-glucose cotransporter 2; T1D, type 1 diabetes; T2D, type 2 diabetes; UACR, urine albumin-to-creatinine ratio; ULN, upper limit of normal. ^a^Patients could have met multiple exclusion criteria.

Of the included 13 799 patients with T2D and CKD stages 1 to 3, 12 331 (89.4%) received SGLT2 inhibitors (7548 men [61.2%] and 4783 women [38.8%]; mean [SD] age, 62.4 [12.3] years; median [IQR] HbA_1c_ level, 8.4% [7.5%-9.7%]; median [IQR] eGFR, 69.0 [53.0-90.7] mL/min/1.73 m^2^; and median [IQR] UACR, 101.0 [38.5-316.5] mg/g), very similar to the GLP-1 RA group (1468 patients; 900 men [61.3%] and 568 women [38.7%]; mean [SD] age, 61.5 [13.3] years) after applying the study inclusion and exclusion criteria and propensity scores with fine stratification weights (Figure 1). Table 1 and eFigure 2 in Supplement 1 present baseline characteristics of the study cohort and propensity score distributions before and after applying propensity scores with fine stratification weights, respectively.

### Main Outcomes

**Figure 2.**
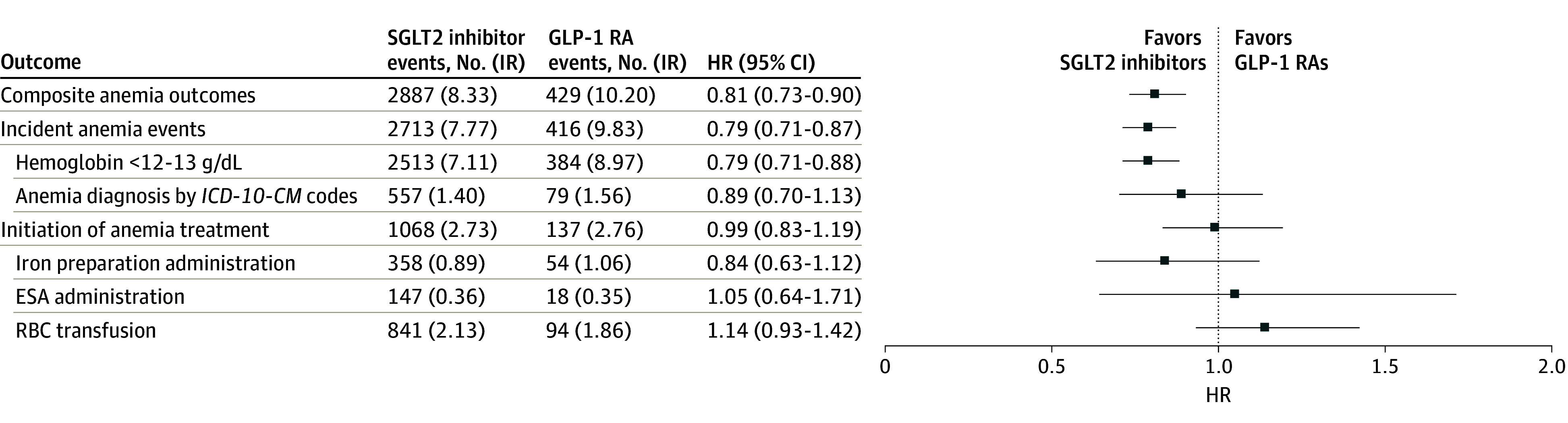
Composite Anemia Outcomes of Sodium-Glucose Cotransporter 2 (SGLT2) Inhibitors and Glucagon-Like Peptide-1 Receptor Agonists (GLP-1 RAs) After Applying Propensity-Score Fine Stratification Weights Incidence rates (IRs) are presented per 100 person-years. To convert hemoglobin to grams per liter, multiply by 10. ESA indicates erythropoiesis-stimulating agents; HR, hazard ratio; *ICD-10-CM*, *International Classification of Diseases, 10th Revision, Clinical Modification*; RBC, red blood cell.

During the median (IQR) follow-up of 2.5 (1.3-4.1) years, we observed 2887 and 429 composite anemia outcomes in patients receiving SGLT2 inhibitors and GLP-1 RAs, respectively. The IR of composite anemia outcome was lower in patients receiving SGLT2 inhibitors (8.33 [95% CI, 8.03-8.64] per 100 person-years) than in those receiving GLP-1 RAs (10.20 [95% CI, 9.26-11.21] per 100 person-years), yielding an HR of 0.81 (95% CI, 0.73-0.90) (eFigure 3 in Supplement 1). The number needed to treat for composite anemia outcome was estimated at 55. Specifically, SGLT2 inhibitors were associated with a reduced risk for incident anemia events (HR, 0.79; 95% CI, 0.71-0.87) but not for initiation of anemia treatment (HR, 0.99; 95% CI, 0.83-1.19) (Figure 2).

### Changes in Hematological Parameters, Glycemic Control, and Kidney Function

The median (IQR) hemoglobin level (13.8 [12.6-15.0] g/dL), hematocrit level (41.0% [37.9%-44.3%] [to convert to proportion of 1.0, multiply by 0.01]), and red blood cell counts (4.7 [4.3-5.1] × 10^6^/μL [to convert to cells × 10^12^/L, multiply by 1]) in the SGLT2 inhibitor group were similar to those in the GLP-1 RA group at baseline. We found these hematological parameters generally remained unchanged in the SGLT2 inhibitor group, while they decreased in the GLP-1 RA group during different follow-up periods (Table 2).

**Table 2.** Changes in Hematological Parameters, Glycemic Control, and Kidney Function After Applying Propensity-Score Fine Stratification Weights

Time period	SGLT2 inhibitors (n = 12 331)		GLP-1 RAs (n = 1468)		SMD^a^
	Patients, No. (%)	Median (IQR)	Patients, No. (%)	Median (IQR)	
**Hematological parameters**					
Hemoglobin, g/dL					
Baseline	7647 (62.0)	13.8 (12.6 to 15.0)	879 (59.9)	13.9 (12.6 to 14.8)	0.03
First year	3885 (31.5)	14.1 (12.7 to 15.4)	508 (34.6)	13.6 (12.1 to 14.8)	0.27
Second year	3068 (24.9)	14.0 (12.5 to 15.3)	409 (27.9)	13.3 (12.2 to 14.5)	0.21
Third year	2322 (18.8)	13.9 (12.2 to 15.3)	327 (22.3)	13.1 (11.9 to 14.4)	0.22
Hematocrit, %					
Baseline	5533 (44.9)	41.0 (37.9 to 44.3)	735 (50.1)	41.5 (38.1 to 44.5)	−0.01
First year	4536 (36.8)	42.1 (38.2 to 45.9)	663 (45.2)	40.7 (36.5 to 43.8)	0.30
Second year	3585 (29.1)	41.8 (37.3 to 45.8)	549 (37.4)	40.4 (36.9 to 43.4)	0.21
Third year	2638 (21.4)	41.4 (36.7 to 45.7)	439 (29.9)	39.2 (36.2 to 43.3)	0.20
RBC count, ×10^6^/μL					
Baseline	4771 (38.7)	4.7 (4.3 to 5.1)	606 (41.3)	4.7 (4.3 to 5.1)	−0.06
First year	3731 (30.3)	4.7 (4.2 to 5.2)	524 (35.7)	4.6 (4.1 to 4.9)	0.26
Second year	2969 (24.1)	4.7 (4.1 to 5.2)	434 (29.6)	4.5 (4.2 to 4.9)	0.18
Third year	2178 (17.7)	4.6 (4.1 to 5.2)	341 (23.2)	4.5 (4.1 to 5.0)	0.11
**Glycemic controls**					
HbA_1c_, %					
Baseline	12 331 (100.0)	8.4 (7.5 to 9.7)	1468 (100.0)	8.6 (7.6 to 9.7)	0.00
First year	11 155 (90.5)	7.6 (6.9 to 8.5)	1354 (92.2)	7.6 (6.9 to 8.7)	−0.02
Second year	8881 (72.0)	7.6 (6.9 to 8.5)	1138 (77.5)	7.6 (6.9 to 8.7)	−0.07
Third year	6428 (52.1)	7.6 (6.9 to 8.6)	891 (60.7)	7.6 (6.7 to 8.7)	0.04
**Kidney function**					
eGFR, mL/min/1.73 m^2^					
Baseline	12 331 (100.0)	69.0 (53.0 to 90.7)	1468 (100.0)	69.3 (52.1 to 90.0)	0.00
First year	11 159 (90.5)	73.5 (54.7 to 95.8)	1363 (92.8)	73.4 (54.5 to 95.8)	0.00
Second year	8942 (72.5)	72.9 (54.2 to 95.1)	1155 (78.7)	69.5 (52.3 to 93.7)	0.05
Third year	6496 (52.7)	74.0 (54.9 to 95.6)	910 (62.0)	70.5 (51.0 to 95.8)	0.08
UACR, mg/g					
Baseline	11 424 (92.6)	101.0 (38.5 to 316.5)	1379 (93.9)	101.0 (40.0 to 352.8)	0.02
First year	8430 (68.4)	80.7 (27.7 to 288.0)	1074 (73.2)	87.5 (26.8 to 339.2)	−0.05
Second year	6522 (52.9)	83.5 (27.5 to 303.4)	839 (57.2)	93.3 (28.2 to 392.9)	−0.09
Third year	4595 (37.3)	89.8 (29.8 to 344.9)	636 (43.3)	97.2 (28.8 to 359.0)	−0.01

Abbreviations: eGFR, estimated glomerular filtration rate; GLP-1 RAs, glucagon-like peptide-1 receptor agonists; HbA_1c_, hemoglobin A_1c_; RBC, red blood cell; SGLT2, sodium-glucose cotransporter-2; SMD, standardized mean difference; UACR, urine albumin-to-creatinine ratio.

SI conversion factors: To convert HbA_1c_ to proportion of total hemoglobin, multiply by 0.01; hematocrit to proportion of 1.0, multiply by 0.01; hemoglobin to grams per liter, multiply by 10; RBC to cells × 10^12^ per liter, multiply by 1.

^a^ An SMD between −0.1 and 0.1 indicates a negligible statistical difference between the 2 treatment groups, and comparisons between the groups were based on complete data analyses.

The median (IQR) HbA_1c_ level (8.4% [7.5%-9.75%]), eGFR (69.0 [53.0-90.7] mL/min/1.73 m^2^) and UACR (101.0 [38.5-316.5] mg/g) in the SGLT2 inhibitor group were also similar to those in the GLP-1 RA group at baseline after applying propensity scores with fine stratification weights. However, we observed no differences in glycemic control and kidney function changes between the 2 treatment groups during different follow-up periods (Table 2).

### Subgroup and Sensitivity Analysis

Results from the subgroup analyses suggested that the lower risks of composite anemia outcomes with SGLT2 inhibitor use generally resembled those from the main analysis (Table 3). Across different CKD stages, we found SGLT2 inhibitors had consistently lower risk of composite anemia outcomes than GLP-1 RAs. Moreover, we found that different individual SGLT2 inhibitors had similar associations with the risk of composite anemia outcomes, including empagliflozin (HR, 0.80; 95% CI, 0.72-0.89), dapagliflozin (HR, 0.80; 95% CI, 0.72-0.90) and canagliflozin (HR, 0.76; 95% CI, 0.64-0.90) (eTable 9 in Supplement 1). The results were consistent throughout 3 sensitivity analyses, including on-treatment analyses (HR, 0.66; 95% CI, 0.58-0.74), analyses excluding patients receiving lixisenatide (HR, 0.75; 95% CI, 0.68-0.83), and analyses excluding patients with remote anemia events (HR, 0.84; 95% CI, 0.75-0.95) (eTable 10 in Supplement 1).

**Table 3.** Results of Subgroup Analyses After Applying Propensity-Score Fine Stratification Weights

Subgroup	Patients, No.	IR (95% CI), per 100 person-years	HR (95% CI)	*P* value for interaction
**Age, y**				
≥65				.29
SGLT2 inhibitors	5514	11.65 (11.09-12.23)	0.82 (0.72-0.94)	
GLP-1 RAs	680	14.14 (12.43-16.03)	1 [Reference]	
≥18 to <65				
SGLT2 inhibitors	6518	6.02 (5.68-6.37)	0.80 (0.68-0.93)	
GLP-1 RAs	783	7.55 (6.49-8.73)	1 [Reference]	
**Sex**				
Male				.51
SGLT2 inhibitors	7631	7.54 (7.18-7.92)	0.79 (0.69-0.92)	
GLP-1 RAs	760	9.49 (8.23-10.89)	1 [Reference]	
Female				
SGLT2 inhibitors	4444	9.59 (9.05-10.15)	0.82 (0.71-0.95)	
GLP-1 RAs	697	11.56 (10.11-13.15)	1 [Reference]	
**HbA_1c_**, %				
≥8.0				.37
SGLT2 inhibitors	7631	8.54 (8.15-8.94)	0.83 (0.74-0.92)	
GLP-1 RAs	1243	10.37 (9.35-11.48)	1 [Reference]	
≥6.5 to <8.0				
SGLT2 inhibitors	3782	8.13 (7.57-8.72)	0.69 (0.54-0.87)	
GLP-1 RAs	224	11.47 (9.01-14.41)	1 [Reference]	
**eGFR, mL/min/1.73 m^2^**				
≥90				.08
SGLT2 inhibitors	2805	4.78 (4.35-5.25)	0.78 (0.61-0.99)	
GLP-1 RAs	372	6.20 (4.87-7.77)	1 [Reference]	
≥60 to <90				
SGLT2 inhibitors	4156	7.25 (6.78-7.74)	0.76 (0.62-0.93)	
GLP-1 RAs	393	9.57 (7.82-11.60)	1 [Reference]	
≥30 to <60				
SGLT2 inhibitors	4588	12.63 (11.97-13.30)	0.73 (0.64-0.83)	
GLP-1 RAs	708	17.17 (15.26-19.24)	1 [Reference]	

Abbreviations: eGFR, estimated glomerular filtration rate; GLP-1 RAs, glucagon-like peptide-1 receptor agonists; HbA_1c_, hemoglobin A_1c_; HR, hazard ratio; IR, incidence rate; SGLT2, sodium-glucose cotransporter 2.

SI conversion factor: To convert HbA_1c_ to proportion of total hemoglobin, multiply by 0.01.

## Discussion

This retrospective cohort study emulating the CREDENCE and DAPA-CKD trials suggested that, compared with GLP-1 RA use, SGLT2 inhibitor use was associated with a 19% lower risk of composite anemia outcomes. The number needed to treat for the composite anemia outcome was estimated at 55; that is, 1 additional composite anemia outcome may be prevented among every 55 patients with T2D and CKD stages 1 to 3 who newly receive SGLT2 inhibitors for a 1-year treatment compared with GLP-1 RA use. More importantly, the findings were consistent across different individual SGLT2 inhibitors, suggesting a class effect of anemia benefits from SGLT2 inhibitors. These main findings were supported by changes in hematological laboratory parameters, which showed significant differences between the SGLT2 inhibitor and GLP-1 RA groups throughout the 3-year follow-up. Furthermore, the similarity in glycemic control and kidney function changes for both the SGLT2 inhibitor and GLP-1 RA groups implied that mechanisms possibly underlying the lower incidence of anemia from SGLT2 inhibitor use are independent of these factors.

The results derived from post hoc analyses of the CREDENCE and DAPA-CKD trials should be viewed primarily as a means to generate hypotheses.^55^ However, the perspective offered by these post hoc analyses is still crucial, given the increased likelihood of chance findings, a consequence of conducting multiple analyses.^55^ Even though our main findings were generally consistent with those reported from post hoc analyses of the CREDENCE and DAPA-CKD trials,^14,15^ the incident anemia risk reductions were smaller (approximately 20%) than those reported by these trials (50%-60%). In addition, in contrast to the results from post hoc analyses of those clinical trials, we found no potential benefits of SGLT2 inhibitors as regards the initiation of anemia treatments. Possible explanations may involve differences in the study eligibility and comparison groups, whereby our findings may be more relevant for clinical practice. First, while the CREDENCE and DAPA-CKD trials recruited participants with a UACR exceeding 300 to 5000 mg/g and 200 to 5000 mg/g,^46,47^ respectively, it has also been reported that fewer than 10% of patients receiving SGLT2 inhibitors present with UACR levels greater than 300 mg/g in kidney care clinics in British Columbia.^56^ By way of contrast, our study specifically included patients with milder proteinuria levels (eg, UACR >30 mg/g) in order to evaluate the outcomes of SGLT2 inhibitors in a wider T2D with CKD population. Second, while previous post hoc analyses from the CREDENCE and DAPA-CKD trials indicated anemia benefits after canagliflozin and dapagliflozin treatment in comparison with a placebo group, it must be noted that the amelioration of HbA_1c_, eGFR, and UACR levels may also independently modify the risk of anemia.^57,58^ Since our study evaluated anemia incidence by comparing to GLP-1 RA use, which produced similar changes in HbA_1c_, eGFR, and UACR levels, the findings from our active-comparator design may well reveal the true anemia effects from SGLT2 inhibitor use, arising through mechanisms other than the improvement of glycemic control and kidney function associated with treatment intensification.

Previous studies have proven that specific patient characteristics may modify the treatment outcomes of SGLT2 inhibitors in patients with T2D. For example, findings from a meta-analysis of clinical trials^59^ have indicated greater benefits with regard to cardiovascular events and kidney outcomes from SGLT2 inhibitors in patients with reduced eGFR levels compared with their counterparts. However, based on our subgroup analyses, we found a consistent reduction in anemia risk from SGLT2 inhibitor treatment in patients across different levels of kidney function, and this observation was in line with the results from the post hoc analyses of the CREDENCE and DAPA-CKD trials.^14,15^ Altogether, these findings may reinforce the proposed mechanism of anemia improvement from SGLT2 inhibitor use through regulation of the hypoxia-inducible factor system and stimulation of EPO secretion.^16,60^ Future studies are warranted to confirm our observations and prove this hypothesis.

### Strengths and Limitations

This study has strengths. To our knowledge, our study was the first large-scale retrospective cohort study emulating the CREDENCE and DAPA-CKD trials to compare the incidence of anemia between SGLT2 inhibitor use and GLP-1 RA use in patients with T2D and CKD. Notably, we modified the inclusion criteria from the CREDENCE and DAPA-CKD trials to increase the generalizability of our findings to a wider CKD population (ie, stages 1-3) and to investigate the potential class effect of SGLT2 inhibitors with regard to incident anemia.

However, we acknowledge several limitations to this study. First, residual confounding may be a concern whenever observational data are analyzed. We attempted to eliminate this bias through adjustment for multiple potential confounders, especially for glycemic levels and kidney functions, such as HbA_1c_, eGFR, and UACR levels to account for the severity of T2D and CKD disease at baseline. Second, because the CGRD only collects EMRs from the largest health care system in Taiwan, the possibility of loss to follow-up or missing data cannot be ignored. However, our study followed the active-comparator design, and such unmeasurable bias would have occurred equally in the SGLT2 inhibitor and GLP-1 RA groups, leaving the relative risk estimates from the treatment comparisons more reliable.^15,61,62^ Third, while the CGRD does not contain comprehensive smoking history information, we included important respiratory diseases, including chronic obstructive pulmonary disease, associated with smoking exposure, in the propensity-score model to mitigate their impacts on our findings. Fourth, the validity of *ICD-9-CM* and *ICD-10-CM* anemia codes in the CGRD has not been verified, so misclassification of outcomes may have occurred. To increase the validity of our outcome definitions, we have also included objective hematological parameters (eg, hemoglobin levels <12-13 g/dL) in the study outcome definition, further reducing the likelihood of bias in our results. Furthermore, as regards the individual GLP-1 RAs, we found their patient numbers were quite limited, especially for lixisenatide and semaglutide, which have been reimbursed for T2D management in Taiwan since 2019 and 2020, respectively. Future studies comparing the associations of SGLT2 inhibitors and individual GLP-1 RAs with anemia outcomes in patients with T2D and CKD are suggested to replicate our findings.

## Conclusions

In this retrospective cohort study emulating the CREDENCE and DAPA-CKD trials and based on multi-institutional routine care data from Taiwan, patients with T2D and CKD stages 1 to 3 and newly receiving SGLT2 inhibitors had a lower incidence of composite anemia outcomes compared with those newly receiving GLP-1 RAs. These results were consistent across several different individual SGLT2 inhibitors, possibly suggesting a drug class benefit with regard to anemia events.

## Notes

Supplement 1. **eFigure 1.** Study design diagram **eFigure 2.** Propensity score distributions **eFigure 3.** Survival curves for composite anemia outcomes over time (in days) **eTable 1.** Target trial emulation design framework **eTable 2.** Comparison of eligibility criteria for the CREDENCE and DAPA-CKD trials, and this proposed study **eTable 3.** Details of exclusion criteria **eTable 4.** Disease diagnosis codes for the exclusion criteria **eTable 5.** Disease diagnosis codes to identify baseline comorbidities **eTable 6.** ATC codes to identify co-medications **eTable 7.** ATC codes to identify exposure or comparator drugs **eTable 8.** Composite anemia outcome definitions **eTable 9.** Individual effects of each SGLT2 inhibitor with regard to composite anemia outcomes **eTable 10.** Sensitivity analysis of composite anemia outcomes

Supplement 2.
Data Sharing Statement

## References

[1] Brière M, Diedisheim M, Dehghani L, Dubois-Laforgue D, Larger E. Anaemia and its risk factors and association with treatments in patients with diabetes: a cross-sectional study. Diabetes Metab. 2021;47(1):101164. doi:10.1016/j.diabet.2020.05.006 32461154

[2] New JP, Aung T, Baker PG, . The high prevalence of unrecognized anaemia in patients with diabetes and chronic kidney disease: a population-based study. Diabet Med. 2008;25(5):564-569. doi:10.1111/j.1464-5491.2008.02424.x 18445169

[3] Go AS, Yang J, Tan TC, ; Kaiser Permanente Northern California CKD Outcomes Study. Contemporary rates and predictors of fast progression of chronic kidney disease in adults with and without diabetes mellitus. BMC Nephrol. 2018;19(1):146. doi:10.1186/s12882-018-0942-1 29929484 PMC6014002

[4] Mohanram A, Zhang Z, Shahinfar S, Keane WF, Brenner BM, Toto RD. Anemia and end-stage renal disease in patients with type 2 diabetes and nephropathy. Kidney Int. 2004;66(3):1131-1138. doi:10.1111/j.1523-1755.2004.00863.x 15327408

[5] Mehdi U, Toto RD. Anemia, diabetes, and chronic kidney disease. Diabetes Care. 2009;32(7):1320-1326. doi:10.2337/dc08-0779 19564475 PMC2699743

[6] Ferreira JP, Anker SD, Butler J, . Impact of anaemia and the effect of empagliflozin in heart failure with reduced ejection fraction: findings from EMPEROR-Reduced. Eur J Heart Fail. 2022;24(4):708-715. doi:10.1002/ejhf.2409 34957660 PMC9303456

[7] Docherty KF, Curtain JP, Anand IS, ; DAPA-HF Investigators and Committees. Effect of dapagliflozin on anaemia in DAPA-HF. Eur J Heart Fail. 2021;23(4):617-628. doi:10.1002/ejhf.2132 33615642 PMC11497230

[8] Kliger AS, Foley RN, Goldfarb DS, . KDOQI US commentary on the 2012 KDIGO Clinical Practice Guideline for Anemia in CKD. Am J Kidney Dis. 2013;62(5):849-859. doi:10.1053/j.ajkd.2013.06.008 23891356

[9] Adane T, Getawa S. Anaemia and its associated factors among diabetes mellitus patients in Ethiopia: a systematic review and meta-analysis. Endocrinol Diabetes Metab. 2021;4(3):e00260. doi:10.1002/edm2.260 34277984 PMC8279623

[10] de Boer IH, Khunti K, Sadusky T, . Diabetes management in chronic kidney disease: a consensus report by the American Diabetes Association (ADA) and Kidney Disease: Improving Global Outcomes (KDIGO). Diabetes Care. 2022;45(12):3075-3090. doi:10.2337/dci22-0027 36189689 PMC9870667

[11] McGuire DK, Shih WJ, Cosentino F, . Association of SGLT2 inhibitors with cardiovascular and kidney outcomes in patients with type 2 diabetes: a meta-analysis. JAMA Cardiol. 2021;6(2):148-158. doi:10.1001/jamacardio.2020.4511 33031522 PMC7542529

[12] Giorgino F, Vora J, Fenici P, Solini A. Renoprotection with SGLT2 inhibitors in type 2 diabetes over a spectrum of cardiovascular and renal risk. Cardiovasc Diabetol. 2020;19(1):196. doi:10.1186/s12933-020-01163-9 33222693 PMC7680601

[13] Ku E, Del Vecchio L, Eckardt KU, ; for Conference Participants. Novel anemia therapies in chronic kidney disease: conclusions from a Kidney Disease: Improving Global Outcomes (KDIGO) Controversies Conference. Kidney Int. 2023;104(4):655-680. doi:10.1016/j.kint.2023.05.009 37236424

[14] Koshino A, Schechter M, Chertow Glenn M, . Dapagliflozin and anemia in patients with chronic kidney disease. *NEJM Evidence*. 2023;2(6):EVIDoa2300049. doi:10.1056/EVIDoa2300049 38320128

[15] Oshima M, Neuen BL, Jardine MJ, . Effects of canagliflozin on anaemia in patients with type 2 diabetes and chronic kidney disease: a post-hoc analysis from the CREDENCE trial. Lancet Diabetes Endocrinol. 2020;8(11):903-914. doi:10.1016/S2213-8587(20)30300-4 33065060

[16] Packer M. Mechanisms of enhanced renal and hepatic erythropoietin synthesis by sodium-glucose cotransporter 2 inhibitors. Eur Heart J. 2023;44(48):5027-5035. doi:10.1093/eurheartj/ehad235 37086098 PMC10733737

[17] Yaribeygi H, Maleki M, Nasimi F, Butler AE, Jamialahmadi T, Sahebkar A. Sodium-glucose co-transporter 2 inhibitors and hematopoiesis. J Cell Physiol. 2022;237(10):3778-3787. doi:10.1002/jcp.30851 35951776

[18] Shin JI, Chang AR, Grams ME, ; CKD Prognosis Consortium. Albuminuria testing in hypertension and diabetes: an individual-participant data meta-analysis in a global consortium. Hypertension. 2021;78(4):1042-1052. doi:10.1161/HYPERTENSIONAHA.121.17323 34365812 PMC8429211

[19] Shao SC, Lin YH, Chang KC, . Sodium glucose co-transporter 2 inhibitors and cardiovascular event protections: how applicable are clinical trials and observational studies to real-world patients? BMJ Open Diabetes Res Care. 2019;7(1):e000472. doi:10.1136/bmjdrc-2019-000742 32043472 PMC6954814

[20] ElSayed NA, Aleppo G, Aroda VR, ; on behalf of the American Diabetes Association. 9. Pharmacologic approaches to glycemic treatment: Standards of Care in Diabetes—2023. Diabetes Care. 2023;46(suppl 1):S140-S157. doi:10.2337/dc23-S009 36507650 PMC9810476

[21] Granata A, Maccarrone R, Anzaldi M, . GLP-1 receptor agonists and renal outcomes in patients with diabetes mellitus type 2 and diabetic kidney disease: state of the art. Clin Kidney J. 2022;15(9):1657-1665. doi:10.1093/ckj/sfac069 36003669 PMC9394722

[22] Shao SC, Chan YY, Kao Yang YH, . The Chang Gung Research Database: a multi-institutional electronic medical records database for real-world epidemiological studies in Taiwan. Pharmacoepidemiol Drug Saf. 2019;28(5):593-600. doi:10.1002/pds.4713 30648314

[23] Tsai MS, Lin MH, Lee CP, . Chang Gung Research Database: a multi-institutional database consisting of original medical records. Biomed J. 2017;40(5):263-269. doi:10.1016/j.bj.2017.08.002 29179881 PMC6138604

[24] Liao SC, Shao SC, Lai ECC, Lin SJ, Huang WI, Hsieh CY. Positive predictive value of *ICD-10* codes for cerebral venous sinus thrombosis in Taiwan’s National Health Insurance Claims Database. Clin Epidemiol. 2022;14:1-7. doi:10.2147/CLEP.S335517 35018122 PMC8740620

[25] Wu LY, Shao SC, Liao SC. Positive predictive value of *ICD-10-CM* codes for myocarditis in claims data: a multi-institutional study in Taiwan. Clin Epidemiol. 2023;15:459-468. doi:10.2147/CLEP.S405660 37057126 PMC10086218

[26] Chang C, Liao SC, Shao SC. Positive predictive values of anaphylaxis diagnosis in claims data: a multi-institutional study in Taiwan. J Med Syst. 2023;47(1):97. doi:10.1007/s10916-023-01989-2 37695529

[27] Shao SC, Su YC, Lai ECC, . Association between sodium glucose co-transporter 2 inhibitors and incident glaucoma in patients with type 2 diabetes: a multi-institutional cohort study in Taiwan. Diabetes Metab. 2022;48(1):101318. doi:10.1016/j.diabet.2022.101318 35017100

[28] Shao SC, Chang KC, Lin SJ, . Differences in outcomes of hospitalizations for heart failure after SGLT2 inhibitor treatment: effect modification by atherosclerotic cardiovascular disease. Cardiovasc Diabetol. 2021;20(1):213. doi:10.1186/s12933-021-01406-3 34688282 PMC8542324

[29] Su YC, Shao SC, Lai ECC, . Risk of diabetic macular oedema with sodium-glucose cotransporter-2 inhibitors in type 2 diabetes patients: a multi-institutional cohort study in Taiwan. Diabetes Obes Metab. 2021;23(9):2067-2076. doi:10.1111/dom.14445 34047442

[30] Su YC, Hung JH, Chang KC, . Comparison of sodium-glucose cotransporter 2 inhibitors vs glucagonlike peptide-1 receptor agonists and incidence of dry eye disease in patients with type 2 diabetes in Taiwan. JAMA Netw Open. 2022;5(9):e2232584. doi:10.1001/jamanetworkopen.2022.32584 36136333 PMC9500553

[31] Shao SC, Chang KC, Lin SJ, . Favorable pleiotropic effects of sodium glucose cotransporter 2 inhibitors: head-to-head comparisons with dipeptidyl peptidase-4 inhibitors in type 2 diabetes patients. Cardiovasc Diabetol. 2020;19(1):17. doi:10.1186/s12933-020-0990-2 32050968 PMC7014757

[32] Tsai DHT, Bell JS, Abtahi S, . Cross-regional data initiative for the assessment and development of treatment for neurological and mental disorders. Clin Epidemiol. 2023;15:1241-1252. doi:10.2147/CLEP.S426485 38146486 PMC10749544

[33] Langan SM, Schmidt SA, Wing K, . The reporting of studies conducted using observational routinely collected health data statement for pharmacoepidemiology (RECORD-PE). BMJ. 2018;363:k3532. doi:10.1136/bmj.k3532 30429167 PMC6234471

[34] Mosenzon O, Leibowitz G, Bhatt DL, . Effect of saxagliptin on renal outcomes in the SAVOR-TIMI 53 Trial. Diabetes Care. 2017;40(1):69-76. doi:10.2337/dc16-0621 27797925

[35] Perkovic V, Toto R, Cooper ME, ; CARMELINA investigators. Effects of linagliptin on cardiovascular and kidney outcomes in people with normal and reduced kidney function: secondary analysis of the CARMELINA Randomized Trial. Diabetes Care. 2020;43(8):1803-1812. doi:10.2337/dc20-0279 32444457 PMC7372065

[36] Committee ADAPP; American Diabetes Association Professional Practice Committee. 10. Cardiovascular disease and risk management: Standards of Medical Care in Diabetes—2022. Diabetes Care. 2022;45(suppl 1):S144-S174. doi:10.2337/dc22-S010 34964815

[37] Committee ADAPP; American Diabetes Association Professional Practice Committee. 11. Chronic kidney disease and risk management: Standards of Medical Care in Diabetes—2022. Diabetes Care. 2022;45(suppl 1):S175-S184. doi:10.2337/dc22-S011 34964873

[38] Aroda VR, Billings LK. GLP-1 RA and SGLT2 inhibitors: in harmony for organ protection. J Am Coll Cardiol. 2023;82(6):526-528. doi:10.1016/j.jacc.2023.06.005 37532423

[39] Navaneethan SD, Zoungas S, Caramori ML, . Diabetes management in chronic kidney disease: synopsis of the KDIGO 2022 clinical practice guideline update. Ann Intern Med. 2023;176(3):381-387. doi:10.7326/M22-2904 36623286

[40] American Diabetes Association Professional Practice Committee. 9. Pharmacologic approaches to glycemic treatment: Standards of Care in Diabetes—2024. Diabetes Care. 2024;47(suppl 1):S158-S178. doi:10.2337/dc24-S009 38078590 PMC10725810

[41] Ueda P, Svanström H, Melbye M, . Sodium glucose cotransporter 2 inhibitors and risk of serious adverse events: nationwide register based cohort study. BMJ. 2018;363:k4365. doi:10.1136/bmj.k4365 30429124 PMC6233755

[42] Engström A, Wintzell V, Melbye M, . Sodium-glucose cotransporter 2 inhibitor treatment and risk of atrial fibrillation: Scandinavian cohort study. Diabetes Care. 2023;46(2):351-360. doi:10.2337/dc22-0714 36508322

[43] Kristensen KB, Henriksen DP, Hallas J, Pottegård A, Lund LC. Sodium-glucose cotransporter 2 inhibitors and risk of nephrolithiasis. Diabetologia. 2021;64(7):1563-1571. doi:10.1007/s00125-021-05424-4 33715024

[44] Levey AS, Inker LA, Coresh J. “Should the definition of CKD be changed to include age-adapted GFR criteria?”: con: the evaluation and management of CKD, not the definition, should be age-adapted. Kidney Int. 2020;97(1):37-40. doi:10.1016/j.kint.2019.08.032 31901355

[45] Xie Q, Hao CM, Ji L, . ACEI/ARB underused in patients with type 2 diabetes in Chinese population (CCMR-3B study). PLoS One. 2015;10(2):e0116970. doi:10.1371/journal.pone.0116970 25675409 PMC4326276

[46] Heerspink HJL, Stefánsson BV, Correa-Rotter R, ; DAPA-CKD Trial Committees and Investigators. Dapagliflozin in patients with chronic kidney disease. N Engl J Med. 2020;383(15):1436-1446. doi:10.1056/NEJMoa2024816 32970396

[47] Perkovic V, Jardine MJ, Neal B, ; CREDENCE Trial Investigators. Canagliflozin and renal outcomes in type 2 diabetes and nephropathy. N Engl J Med. 2019;380(24):2295-2306. doi:10.1056/NEJMoa1811744 30990260

[48] Desai RJ, Franklin JM. Alternative approaches for confounding adjustment in observational studies using weighting based on the propensity score: a primer for practitioners. BMJ. 2019;367:l5657. doi:10.1136/bmj.l5657 31645336

[49] Desai RJ, Rothman KJ, Bateman BT, Hernandez-Diaz S, Huybrechts KF. A propensity-score-based fine stratification approach for confounding adjustment when exposure is infrequent. Epidemiology. 2017;28(2):249-257. doi:10.1097/EDE.0000000000000595 27922533 PMC5497217

[50] Pradhan R, Lu S, Yin H, . Novel antihyperglycaemic drugs and prevention of chronic obstructive pulmonary disease exacerbations among patients with type 2 diabetes: population based cohort study. BMJ. 2022;379:e071380. doi:10.1136/bmj-2022-071380 36318979 PMC9623550

[51] Xie Y, Bowe B, Gibson AK, . Comparative effectiveness of SGLT2 inhibitors, GLP-1 receptor agonists, DPP-4 inhibitors, and sulfonylureas on risk of kidney outcomes: emulation of a target trial using health care databases. Diabetes Care. 2020;43(11):2859-2869. doi:10.2337/dc20-1890 32938746

[52] Wang CY, Wu YL, Sheu WH, Tu ST, Hsu CC, Tai TY. Accountability and utilization of diabetes care from 2005 to 2014 in Taiwan. J Formos Med Assoc. 2019;118(suppl 2):S111-S121. doi:10.1016/j.jfma.2019.08.010 31590971

[53] Tsai DHT, Chang WH, Lin HW, Lin SJ, Shao SC, Lai ECC. Post-discharge use of antipsychotics in patients with hospital-acquired delirium and associated risk of mortality—a population-based nested case-control study. Asian J Psychiatr. 2023;83:103533. doi:10.1016/j.ajp.2023.103533 36863305

[54] Pfeffer MA, Claggett B, Diaz R, ; ELIXA Investigators. Lixisenatide in patients with type 2 diabetes and acute coronary syndrome. N Engl J Med. 2015;373(23):2247-2257. doi:10.1056/NEJMoa1509225 26630143

[55] Solomon SD, Pfeffer MA. The future of clinical trials in cardiovascular medicine. Circulation. 2016;133(25):2662-2670. doi:10.1161/CIRCULATIONAHA.115.020723 27324361

[56] Yi TW, Atiquzzaman M, Zheng Y, Smyth B, Jardine M, Levin A. Findings of sodium-glucose cotransporter-2 inhibitor kidney outcome trials applied to a Canadian chronic kidney disease population: a retrospective cohort study. Can J Kidney Health Dis. Published online December 20, 2022. doi:10.1177/20543581221145068 36578697 PMC9791275

[57] Al-Khoury S, Afzali B, Shah N, Covic A, Thomas S, Goldsmith DJ. Anaemia in diabetic patients with chronic kidney disease—prevalence and predictors. Diabetologia. 2006;49(6):1183-1189. doi:10.1007/s00125-006-0254-z 16609874

[58] Aydın B, Özçelik S, Kilit TP, Eraslan S, Çelik M, Onbaşı K. Relationship between glycosylated hemoglobin and iron deficiency anemia: a common but overlooked problem. Prim Care Diabetes. 2022;16(2):312-317. doi:10.1016/j.pcd.2022.01.002 35000894

[59] Chun KJ, Jung HH. SGLT2 inhibitors and kidney and cardiac outcomes according to estimated GFR and albuminuria levels: a meta-analysis of randomized controlled trials. Kidney Med. 2021;3(5):732-744.e1. doi:10.1016/j.xkme.2021.04.009 34746739 PMC8551546

[60] Packer M. Role of impaired nutrient and oxygen deprivation signaling and deficient autophagic flux in diabetic CKD development: implications for understanding the effects of sodium-glucose cotransporter 2-inhibitors. J Am Soc Nephrol. 2020;31(5):907-919. doi:10.1681/ASN.2020010010 32276962 PMC7217421

[61] Fu EL, van Diepen M, Xu Y, . Pharmacoepidemiology for nephrologists (part 2): potential biases and how to overcome them. Clin Kidney J. 2020;14(5):1317-1326. doi:10.1093/ckj/sfaa242 33959262 PMC8087121

[62] Prada-Ramallal G, Takkouche B, Figueiras A. Bias in pharmacoepidemiologic studies using secondary health care databases: a scoping review. BMC Med Res Methodol. 2019;19(1):53. doi:10.1186/s12874-019-0695-y 30871502 PMC6419460

