# Supplementary material for: Use of SGLT2 Inhibitors vs GLP-1 RAs and Anemia in Patients With Diabetes and CKD
Source: JAMA Netw Open. 2024 Mar 4;7(3):e240946. doi: 10.1001/jamanetworkopen.2024.0946 (PMC10912959; doi:10.1001/jamanetworkopen.2024.0946)
Supplement: Supplement 1. — eFigure 1. Study design diagram eFigure 2. Propensity score distributions eFigure 3. Survival curves for composite anemia outcomes over time (in days) eTable 1. Target trial emulation design framework eTable 2. Comparison of eligibility criteria for the CREDENCE and DAPA-CKD trials, and this proposed study eTable 3. Details of exclusion criteria eTable 4. Disease diagnosis codes for the exclusion criteria eTable 5. Disease diagnosis codes to identify baseline comorbidities eTable 6. ATC codes to identify co-medications eTable 7. ATC codes to identify exposure or comparator drugs eTable 8. Composite anemia outcome definitions eTable 9. Individual effects of each SGLT2 inhibitor with regard to composite anemia outcomes eTable 10. Sensitivity analysis of composite anemia outcomes [file jamanetwopen-e240946-s001.pdf]

Supplemental Online Content

Hu JC, Shao SC, Tsai DHT, Chuang ATM, Liu KH, Lai ECC. Use of SGLT2 inhibitors vs GLP-1 RAs and anemia in patients with diabetes and CKD. *JAMA Netw Open.* 2024;7(3):e240946.
doi:10.1001/jamanetworkopen.2024.0946

eFigure 1. Study design diagram

eFigure 2. Propensity score distributions

eFigure 3. Survival curves for composite anemia outcomes over time (in days)

eTable 1. Target trial emulation design framework

eTable 2. Comparison of eligibility criteria for the CREDENCE and DAPA-CKD trials, and this proposed study

eTable 3. Details of exclusion criteria

eTable 4. Disease diagnosis codes for the exclusion criteria

eTable 5. Disease diagnosis codes to identify baseline comorbidities

eTable 6. ATC codes to identify co-medications

eTable 7. ATC codes to identify exposure or comparator drugs

eTable 8. Composite anemia outcome definitions

eTable 9. Individual effects of each SGLT2 inhibitor with regard to composite anemia outcomes

eTable 10. Sensitivity analysis of composite anemia outcomes

This supplemental material has been provided by the authors to give readers additional information about their work.

© 2024 Hu JC et al. *JAMA Network Open.*

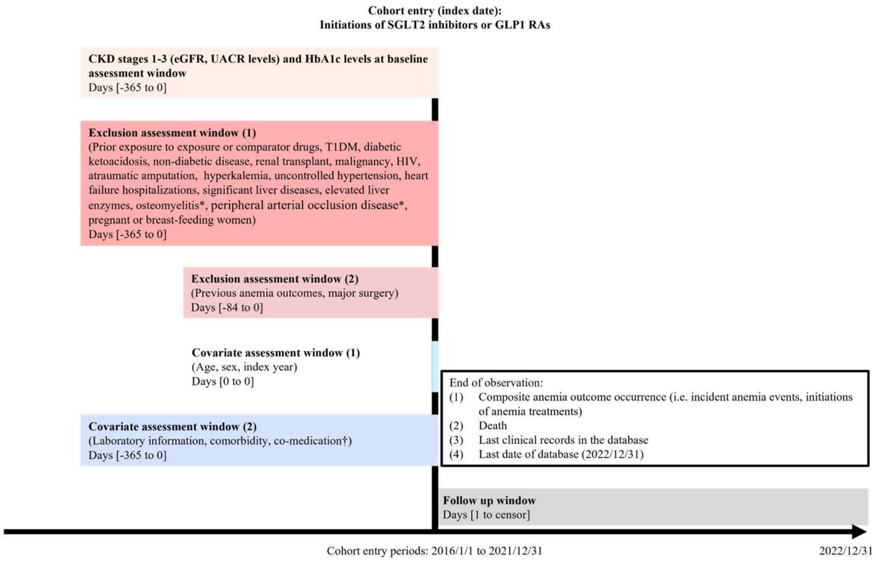

eFigure 1. Study design diagram

CKD: Chronic kidney disease; eGFR: Estimated glomerular filtration rate; GLP-1 RAs: Glucagon-like peptide-1 receptor agonists; HbA1c: Hemoglobin A1C; HIV: human immunodeficiency virus; SGLT2: Sodium-glucose cotransporter-2; T1DM: Type 1 diabetes mellitus; UACR: Urine albumin-to-creatinine ratio

* Osteomyelitis, and peripheral arterial occlusion disease were considered within 6 months.

† Co-medications only included drugs that were prescribed 84 days before the index date.

© 2024 Hu JC et al. *JAMA Network Open.*

(a) Original cohort

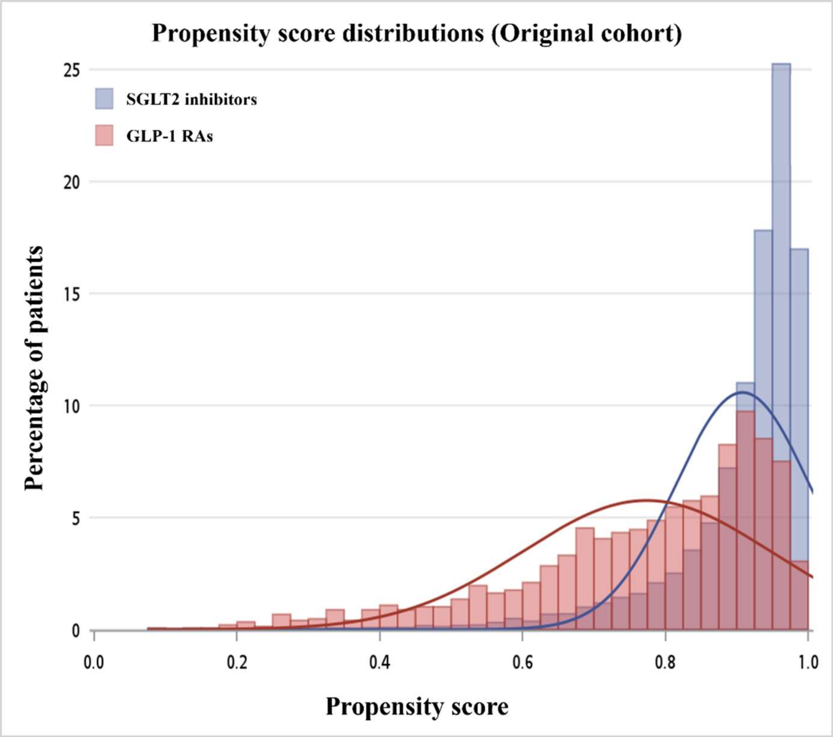

(b) Weighted cohort

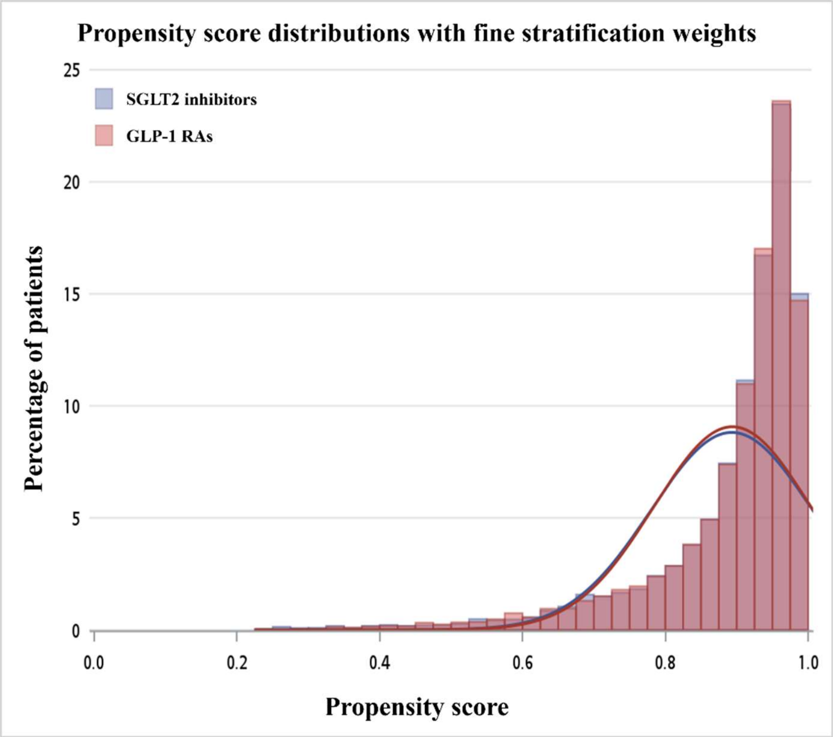

eFigure 2. Propensity score distributions

© 2024 Hu JC et al. *JAMA Network Open.*

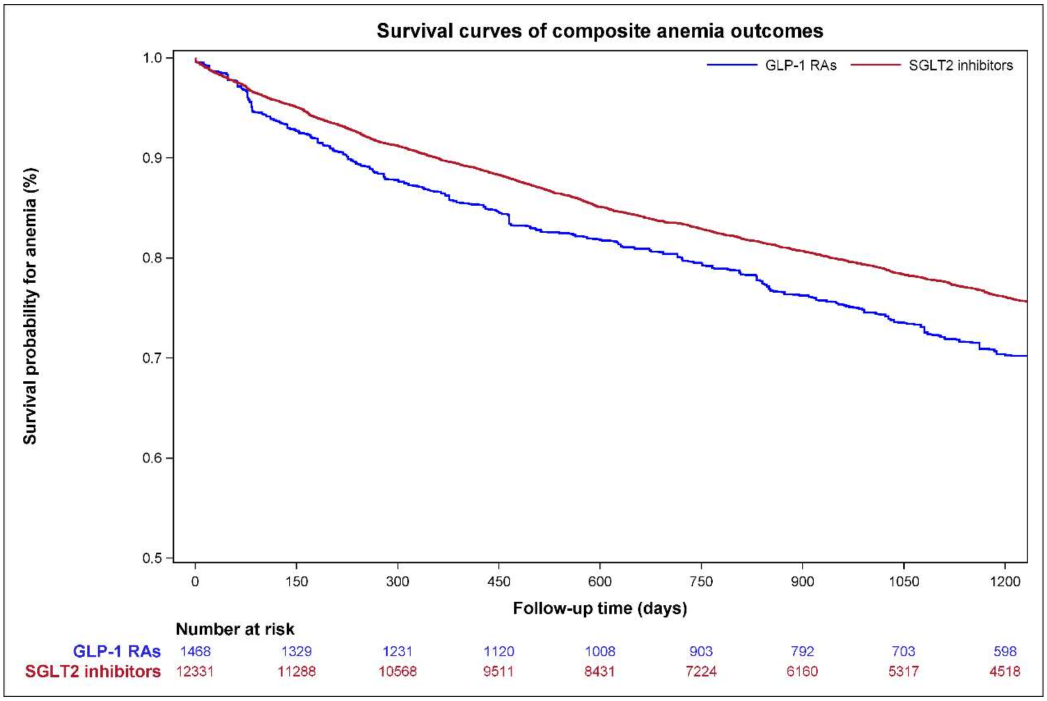

eFigure 3. Survival curves for composite anemia outcomes over time (in days)
Abbreviations: GLP-1 RAs: Glucagon-like peptide-1 receptor agonists; SGLT2: Sodium-glucose cotransporter-2

© 2024 Hu JC et al. *JAMA Network Open.*

eTable 1. Target trial emulation design framework

Protocol component	Target trial, modified from the CREDENCE and DAPA-CKD trials for anemia outcomes	Proposed study
Study aim	To assess the effects of SGLT2 inhibitors on anemia-related outcomes in participants with T2DM and CKD.	Same as target trial.
Study design	Open-labelled, pragmatic clinical trial.	Same as target trial.
Eligibility criteria [Inclusion]	1. Participants ≥ 18 years old with T2DM.<br>2. HbA1c levels more than 6.5% in the baseline period.<br>3. CKD stage 3 (eGFR ≥30 to <60 ml/min/1.73 m^2) or CKD stages 1-2 with proteinuria (UACR ≥ 30 mg/g) in the baseline period.	Same as target trial.
Eligibility criteria [Exclusion]	1. History of use of exposure- or comparator drugs.<br>2. History of diabetic ketoacidosis or T1DM.<br>3. History of hereditary glucose-galactose malabsorption, primary renal glucosuria.<br>4. History of hereditary nephropathy, primary nephrotic syndrome.<br>5. History of renal transplant.<br>6. Blood potassium level > 5.5 mmol/l twice in the last two records in the baseline period.<br>7. Uncontrolled hypertension (SBP ≥ 180 and/or DBP ≥ 100 mmHg twice in the last	Same as target trial

© 2024 Hu JC et al. *JAMA Network Open.*

two records in the baseline period).

8. Clinical diagnosis of hospitalizations for heart failure at discharge with BNP levels > 400 ρg/ml in the baseline period.
9. Clinical diagnosis of significant liver disease (including hepatic failure, chronic hepatitis, fibrosis, and liver cirrhosis) in the baseline period.
10. ALT levels >2.0 times the ULN or total bilirubin >1.5 times the ULN twice in the last two records in the baseline period.
11. Participants who had a clinical diagnosis of malignancy in the baseline period.
12. History of human immunodeficiency virus.
13. Major surgery (defined as hospitalization record with blood transfusion history) within 12 weeks before the index date.
14. History of atraumatic amputation.
15. Clinical diagnosis of osteomyelitis, or peripheral arterial occlusion disease within 6 months in the baseline period.

© 2024 Hu JC et al. *JAMA Network Open.*

	16. Pregnant or breast-feeding women in the baseline period.<br>17. Participants with previous anemia outcomes within 12 weeks before the index date.	
Treatment strategies	1. SGLT2 inhibitors (empagliflozin, dapagliflozin, canagliflozin and ertugliflozin) or<br>2. GLP-1 RAs (lixisenatide, liraglutide, dulaglutide and semaglutide)	Same as target trial.
Treatment assignment	Randomly allocated to receive SGLT2 inhibitors or GLP-1 RAs in order to generate two groups with same probability of treatment assignment.	Propensity score method with fine stratification to generate two groups with similar probability of treatment assignment.
Outcomes	1. Composite anemia outcomes:<br>(1) Incident anemia events:<br>• Hemoglobin less than 13 g/dl in men or less than 12 g/dl in women after the index date.<br>• Anemia diagnoses by ICD-10-CM codes.<br>(2) Initiation of anemia treatments (iron preparations, erythropoiesis-stimulating agents or red blood cell transfusion).<br>2. Hematological parameter changes (hemoglobin,	Same as target trial.

© 2024 Hu JC et al. *JAMA Network Open.*

	hematocrit and red blood cell count).	
Follow up	Follow-up until the end of the trial.	The end of observations was set as:<br>• Occurrence of composite anemia outcomes<br>• Death<br>• Last clinical records in the database<br>• Last date of database (2022/12/31)
Causal contrasts	Intention to treat effect.	Same as target trial.
Statistical analysis	Kaplan-Meier analysis with Cox proportional hazards models to compare effects on anemia of SGLT2 inhibitors vs. GLP-1 RAs.	Same as target trial, and we further conducted:<br>1. Subgroup analysis based on age, sex, HbA1c levels, eGFR levels and individual SGLT2 inhibitors.<br>2. Sensitivity analysis of on-treatment (as-treated) analysis, excluding lixisenatide analysis and excluding remote anemia event analysis.

Abbreviations: ALT: Alanine transaminase; CREDENCE: Canagliflozin and Renal Events in Diabetes with Established Nephropathy Clinical Evaluation; CKD: Chronic kidney disease; CI: Confidence interval; DAPA-CKD: Dapagliflozin in Patients with Chronic Kidney Disease; DBP: Diastolic blood pressure; T1DM: Type 1 diabetes mellitus; T2DM: Type 2 diabetes mellitus; eGFR: Estimated glomerular filtration rate; GLP-1 RAs: Glucagon-like peptide-1 receptor agonists; HbA1c: Hemoglobin A1C; ICD-10-CM: International Classification of Diseases, Tenth Revision, Clinical Modification; SBP: Systolic blood pressure; SGLT2: Sodium-glucose cotransporter-2; ULN: Upper limit normal; UACR: Urine albumin-to-creatinine ratio

© 2024 Hu JC et al. *JAMA Network Open.*

eTable 2. Comparison of eligibility criteria for the CREDENCE and DAPA-CKD trials, and this proposed study

	CREDENCE trial	DAPA-CKD trial	Target trial, modified from CREDENCE and DAPA-CKD trials for anemia outcomes reference[a]	Proposed study
Inclusion criteria	1. Man or woman ≥ 30 years-old with a clinical diagnosis of type 2 diabetes mellitus.<br>2. HbA1c levels of 6.5–12.0%<br>3. eGFR ≥ 30 to < 90 mL/min/1.73 m^2<br>4. UACR > 300 mg/g to ≤ 5000 mg/g<br>5. All subjects must be on a stable maximum tolerated labeled daily dose of ACE inhibitors or ARB for at least 4 weeks prior to randomization [a]<br>6. Women must be postmenopausal, surgically sterile, heterosexually active and practicing a highly effective method of birth control, not heterosexually active.<br>7. Women of	1. Provision of signed informed consent prior to any study specific procedures [b]<br>2. Female or male aged ≥ 18 years at the time of consent<br>3. eGFR ≥ 25 and ≤ 75 mL/min/1.73m^2 at visit 1<br>4. UACR ≥ 200 and ≤ 5000 mg/g at visit 1<br>5. Stable, and for the patient maximum tolerated labelled daily dose, treatment with ACE inhibitors or ARB for at least 4 weeks before visit 1, if not medically contraindicated. [a]	1. Participants ≥ 18 years old with T2DM. [CREDENCE: #1; DAPA-CKD: #2]<br>2. HbA1c levels more than 6.5% in the baseline period. [CREDENCE: #2]<br>3. CKD stage 3 (eGFR ≥ 30 to < 60 ml/min/1.73 m^2) or CKD stages 1-2 with proteinuria (UACR ≥ 30 mg/g) in the baseline period. [CREDENCE: #3,4; DAPA-CKD: #4]	Same as target trial.

© 2024 Hu JC et al. *JAMA Network Open.*

	childbearing potential must have a negative urine pregnancy test at baseline.<br>8. Willing and able to adhere to the prohibitions and restrictions specified in this protocol [b]<br>9. Subjects must have signed an informed consent document indicating that they understand the purpose of and procedures required for the study and are willing to participate in the study. [b]			
Exclusion criteria	1. History of diabetic ketoacidosis or T1DM.<br>2. History of hereditary glucose-galactose malabsorption or primary renal glucosuria.<br>3. Known medical history or clinical evidence suggesting nondiabetic renal disease.<br>4. Renal disease that required treatment with	1. Autosomal dominant or autosomal recessive polycystic kidney disease, lupus nephritis or ANCA-associated vasculitis.<br>2. Receiving cytotoxic therapy, immunosuppressive therapy or other immunotherapy for primary or secondary renal disease within 6 months prior to	1. History of use of exposure or comparator drugs. [CREDENCE: #19; DAPA-CKD: #4]<br>2. History of diabetic ketoacidosis or T1DM. [CREDENCE: #1; DAPA-CKD:#5]<br>3. History of hereditary glucose-galactose	Same as target trial.

© 2024 Hu JC et al. *JAMA Network Open.*

	immunosuppressive therapy or a history of chronic dialysis or renal transplant.<br>5. Uncontrolled hypertension (SBP ≥ 180 and/or DBP ≥ 100 mmHg) by Week 2<br>6. Blood potassium level > 5.5 mmol/L during screening<br>7. MI, unstable angina, revascularization procedure (e.g., stent or bypass graft surgery), or cerebrovascular accident within 12 weeks before randomization [c]<br>8. Heart failure of NYHA class IV cardiac disease [d]<br>9. Electrocardiogram findings within 12 weeks before randomization that would require urgent diagnostic evaluation or intervention [c]<br>10. Significant liver disease (e.g., acute hepatitis, chronic active hepatitis, cirrhosis)	enrolment.<br>3. History of organ transplantation.<br>4. Receiving therapy with an SGLT2 inhibitor within 8 weeks prior to enrolment or previous intolerance of an SGLT2 inhibitor.<br>5. T1DM<br>6. NYHA class IV Congestive Heart Failure at the time of enrolment. [d]<br>7. MI, unstable angina, stroke or transient ischemic attack within 12 weeks prior to enrolment. [c]<br>8. Coronary revascularization (percutaneous coronary intervention or coronary artery bypass grafting) or valvular repair/replacement within 12 weeks prior to enrolment or is planned to undergo any of these procedures after randomization. [c]	malabsorption, primary renal glucosuria. [CREDENCE: #2]<br>4. History of hereditary nephropathy, primary nephrotic syndrome. [CREDENCE: #3; DAPA-CKD: #1,2]<br>5. History of renal transplant. [CREDENCE: #4; DAPA-CKD: #2,3]<br>6. Blood potassium level >5.5 mmol/l twice in the last two records in the baseline period. [CREDENCE: #6]<br>7. Uncontrolled hypertension (SBP ≥ 180 and/or DBP ≥ 100 mmHg twice in the last two records in the	

© 2024 Hu JC et al. *JAMA Network Open*.

	11. ALT levels > 2.0 times the ULN or total bilirubin > 1.5 times the ULN<br>12. History of malignancy within 5 years before screening<br>13. History of HIV antibody positive<br>14. Major surgery within 12 weeks before randomization [c]<br>15. Any condition that in the opinion of the investigator or sponsor's medical monitor would make participation not in the best interest of the subject, or could prevent, limit, or confound the protocol specified assessments [b]<br>16. History of atraumatic amputation within the past 12 months of screening, or an active skin ulcer, osteomyelitis, gangrene, or critical ischemia of the lower extremity within 6 months of	9. Any condition outside the renal and CV disease area, such as but not limited to malignancy, with a life expectancy of less than 2 years based on investigator´s clinical judgement. [b]<br>10. Active malignancy requiring treatment at the time of visit 1 (with the exception of successfully treated basal cell or treated squamous cell carcinoma).<br>11. Hepatic impairment (AST or ALT >3 times the ULN; or total bilirubin >2 times ULN at time of enrolment).<br>12. Known blood-borne diseases such as Ebola, Lassa fever virus, Hepatitis A, B, C, D, and E viruses, HIV types 1 and 2.[g]<br>13. Women of child-bearing potential (e.g., those who are not chemically or	baseline period). [CREDENCE: #5]<br>8. Clinical diagnosis of hospitalizations for heart failure at discharge with BNP levels > 400 ρg/ml in the baseline period. [d] [CREDENCE: #8; DAPA-CKD: #6]<br>9. Clinical diagnosis of significant liver disease (including hepatic failure, chronic hepatitis, fibrosis, and liver cirrhosis) in the baseline period. [CREDENCE: #10]<br>10. ALT levels > 2.0 times the ULN or total bilirubin > 1.5 times the ULN twice in the last two	

© 2024 Hu JC et al. *JAMA Network Open.*

	screening.<br>17. Combination use of an ACE inhibitor and ARB. [e]<br>18. Use of a mineralocorticoid-receptor antagonist or a direct renin inhibitor [f]<br>19. Current use of an SGLT2 inhibitor, within 12 weeks prior to randomization.<br>20. Current participation in another canagliflozin study or previously exposed to canagliflozin in a prior canagliflozin study. [b]<br>21. Known allergies, hypersensitivity, or intolerance to canagliflozin or its excipients. [b]<br>22. Received an active investigational drug (including vaccines) other than a placebo agent, or used an investigational medical device within 12 weeks before Day	surgically sterilized or who are not post-menopausal) who are not willing to use a medically accepted method of contraception that is considered reliable in the judgment of the investigator OR women who have a positive pregnancy test at enrolment or randomization OR women who are breast-feeding.<br>14. Involvement in the planning and/or conduct of the study. [b]<br>15. Previous randomization in the present study. [b]<br>16. Participation in another clinical study with an IP during the last month prior to enrolment. [b]<br>17. Inability of the patient, in the opinion of the investigator, to understand and/or comply with IP, procedures and/or follow-up OR any	records in the baseline period. [CREDENCE: #11; DAPA-CKD: #11]<br>11. Participants who had a clinical diagnosis of malignancy in the baseline period. [CREDENCE: #12; DAPA-CKD: #9,10]<br>12. History of HIV. [CREDENCE: #13; DAPA-CKD: #12]<br>13. Major surgery (defined as hospitalization record with blood transfusion history) within 12 weeks before the index date. [c] [CREDENCE: #7,9,14 ; DAPA-CKD: #7,8]<br>14. History of atraumatic amputation.	

© 2024 Hu JC et al. *JAMA Network Open.*

	1/baseline. [b]<br>23. Pregnant or breast-feeding or planning to become pregnant or breast-feed during the study.<br>24. Employees of the investigator or study center, with direct involvement in the proposed study or other studies under the direction of that investigator or study center, as well as family members of the employees or the investigator. [b]	conditions that, in the opinion of the investigator, may render the patient unable to complete the study. [b]	[CREDENCE: #16]<br>15. Clinical diagnosis of osteomyelitis, or peripheral arterial occlusion disease within 6 months in the baseline period. [CREDENCE: #16]<br>16. Pregnant or breast-feeding women in the baseline period. [CREDENCE: inclusion #6,7, #23; DAPA-CKD: #13]<br>17. Participants with previous anemia outcomes within 12 weeks before index date.	

Note: a: The criterion of 'Stable maximum tolerated labeled daily dose of ACE inhibitor or ARB' was removed in the presented target trial emulation study because the CREDENCE and DAPA-CKD trials aimed to assess the effects of SGLT2 inhibitors on renal outcomes in participants at high risk of CKD progression. In addition, underuse of these medications in patients with T2DM in clinical practice has been reported.

b: These criteria are standard requirements in conducting clinical trials, but they were

© 2024 Hu JC et al. *JAMA Network Open.*

not applicable in the observational study.

c: All procedural criteria were condensed into 'hospitalization record with a blood transfusion history within 12 weeks before the index date' with stricter criteria specifically tailored to our study outcomes.

d: As there was no direct NYHA stage classification in the database, severe heart failure was redefined as 'clinical diagnosis of hospitalizations for heart failure at discharge and BNP levels > 400 pg/ml' during the baseline period.

e: According to our reimbursement regulations, the combination use of ACE inhibitors and ARBs is contraindicated and not permitted in our reimbursement plan. Therefore, the combination of these two medications will not occur.

f: The CREDENCE trial focuses on the renal outcomes of SGLT2 inhibitors, while MRA and DRI are potential treatments for diabetic nephropathy. Although anemia-related outcomes were a major objective in the study, it did not exclude patients who took MRA or DRI during the baseline.

g: Ebola and Lassa fever viruses are not prevalent diseases in Taiwan. The exclusion criteria eliminated patients with significant liver disease, which could be the result of hepatitis viruses.

Abbreviations: ACE: Angiotensin-converting enzyme; ALT: Alanine transaminase; ANCA: Anti-neutrophil cytoplasmic antibodies; ARBs: Angiotensin receptor blockers; AST: Aspartate transaminase; BNP, B-type Natriuretic Peptide; CI: Confidence interval; CKD: Chronic kidney disease; CREDENCE: Canagliflozin and Renal Events in Diabetes with Established Nephropathy Clinical Evaluation; CV: Cardiovascular; DAPA-CKD: Dapagliflozin in Patients with Chronic Kidney Disease; DBP: Diastolic blood pressure; e.g.: For example; eGFR: Estimated glomerular filtration rate; GLP-1 RAs: Glucagon-like peptide-1 receptor agonists; HbA1c: Hemoglobin A1C; HIV: Human immunodeficiency virus; ICD-10-CM: International Classification of Diseases, Tenth Revision, Clinical Modification; IP: Investigational Product; MI: myocardial infarction; NYHA: New York Heart Association; SBP: Systolic blood pressure; SGLT2: Sodium-glucose cotransporter-2; T1DM: Type 1 diabetes mellitus; T2DM: Type 2 diabetes mellitus; ULN: Upper limit normal; UACR: Urine albumin-to-creatinine ratio

© 2024 Hu JC et al. *JAMA Network Open.*

eTable 3. Details of exclusion criteria

Patients with at least two diagnosis coding records from inpatient or outpatient electronic medical records data:

1. Patients who had a history of diabetic ketoacidosis or type 1 diabetes mellitus.
2. Patients with a history of non-diabetic kidney diseases, such as hereditary glucose-galactose malabsorption or primary renal glucosuria.
3. Patients with a history of hereditary nephropathy or primary nephrotic syndrome.
4. Patients with a history of renal transplant by at least two coding records.
5. Patients with hyperkalemia, i.e., blood potassium levels exceeding 5.5 mmol/l twice within the year preceding the index date.
6. Patients with uncontrolled hypertension, defined as systolic blood pressure exceeding 180 mmHg or diastolic blood pressure exceeding 100 mmHg, recorded twice within the year preceding the index date.
7. Patients with a clinical diagnosis of severe heart failure, defined as hospitalization for heart failure at discharge with B-type natriuretic peptide levels higher than 400 ρg/ml, within one year before the index date.
8. Patients with elevated liver enzymes, with either alanine transaminase levels more than 2.0 times the upper limit normal (50 U/l in males; 35 U/l in females) or total bilirubin more than 1.5 times the upper limit normal (1.2 mg/dl), twice in the last two results within one year before the index date.
9. Patients with a clinical diagnosis of significant liver disease (including hepatic failure, chronic hepatitis, fibrosis and liver cirrhosis) within one year before the index date.
10. Patients who had a clinical diagnosis of malignancy within one year before the index date.
11. Patients with a history of human immunodeficiency virus.
12. Patients with a history of atraumatic amputation.
13. Patients who underwent major surgery (defined as hospitalization record with red blood cell transfusion history) within 12 weeks before the index date.
14. Patients who had a clinical diagnosis of osteomyelitis or peripheral arterial occlusion disease within six months of the baseline period.
15. Pregnant or breastfeeding women within one year before the index date.
16. Patients who had previous outcome events within 12 weeks before the index date.

© 2024 Hu JC et al. *JAMA Network Open.*

eTable 4. Disease diagnosis codes for exclusion criteria

Disease	ICD-9-CM	ICD-10-CM
Diabetic ketoacidosis	249.1, 250.1	E11.X-E14.X, X=1
Hereditary glucose-galactose malabsorption	271.0, 271.1	E74.2, E74.3
Primary renal glucosuria	271.4	E74.8
Hereditary nephropathy	583	N07
Nephritic syndrome	580-582	N00-N03
Obstructive uropathy	593	N13
Renal transplant	V42.0	Z94.0
Heart failure	428	I50
Significant liver disease	570-572	K72-K74
Malignancy	140-202	C00-C97, D00-D09
Human immunodeficiency virus	042-044, 795.71, 079.53	B20-B24, R75, B97.35
Atraumatic amputation	E878	Y83
Osteomyelitis	730	M86
Peripheral arterial occlusion disease	444.22	I74.4
Pregnant or breastfeeding women	V22.X, X=0,1	Z32-34

Abbreviations: ICD-9-CM: International Classification of Diseases, Ninth Revision, Clinical Modification; ICD-10-CM: International Classification of Diseases, Tenth Revision, Clinical Modification

© 2024 Hu JC et al. *JAMA Network Open.*

eTable 5. Disease diagnosis codes to identify baseline comorbidities

Comorbidity		ICD-9-CM	ICD-10-CM
Heart failure		428	I50
Ischemic heart disease		410-414	I20-I25
Peripheral arterial disease		440, 443	I70, I73
Ischemic stroke		433, 434	I63, I66
Atrial fibrillation		427.3	I48
Hypertension		401-405	I10-I15
Dyslipidemia		272	E78
Autoinflammatory diseases	Inflammatory bowel disease,	555-558	K50-K52
	Systemic lupus erythematosus,	710.0	M32
	Rheumatoid arthritis,	714	M05
	Sarcoidosis	135	D86
Respiratory diseases	Asthma,	493	J45
	Chronic obstructive pulmonary disease,	491, 492, 496	J44
	Pulmonary arterial hypertension	416	I27
Thyroid gland disorders	Hypothyroidism	242	E03
	Hyperthyroidism	244	E05

Abbreviations: ICD-9-CM: International Classification of Diseases, Ninth Revision, Clinical Modification; ICD-10-CM: International Classification of Diseases, Tenth Revision, Clinical Modification

© 2024 Hu JC et al. *JAMA Network Open.*

eTable 6. ATC codes to identify co-medications

Drugs		ATC code
Diabetes medications	Insulin	A10AB01, A10AB04, A10AB05, A10AB06, A10AB30, A10AC01, A10AC04, A10AC30, A10AD01, A10AD05 A10AD06, A10AD30, A10AE01, A10AE04, A10AE05, A10AE06, A10AE30, A10AE54, A10AE56, A10AF01
	Metformin	A10BA02, A10BD02, A10BD03, A10BD05, A10BD07, A10BD08, A10BD10, A10BD11, A10BD13, A10BD14, A10BD15, A10BD16, A10BD17, A10BD18, A10BD20, A10BD22, A10BD23, A10BD25, A10BD26, A10BD27
	Sulfonylurea	A10BB01, A10BB02, A10BB03, A10BB04, A10BB05, A10BB06, A10BB07, A10BB08, A10BB09, A10BB10, A10BB11, A10BB12, A10BB31, A10BC01, A10BD01, A10BD02, A10BD04, A10BD05, A10BD06
	Alpha-glucosidase inhibitor	A10BF01, A10BF02, A10BF03, A10BD17
	Thiazolidinedione	A10BG01, A10BG02, A10BG03, A10BG04, A10BD03, A10BD04, A10BD05, A10BD06, A10BD09, A10BD12, A10BD26
	Dipeptidyl peptidase-4 inhibitor	A10BH01, A10BH02, A10BH03, A10BH04, A10BH05, A10BH06, A10BH07, A10BH08, A10BH51, A10BH52, A10BD07, A10BD08, A10BD09, A10BD10, A10BD11, A10BD12, A10BD13, A10BD19, A10BD21, A10BD22, A10BD24, A10BD25, A10BD27
	Meglitinide	A10BX02, A10BX08, A10BD14
Vitamin B12 or folic acid		B03AE01, B03AE02, B03BA, B03BB
Angiotensin-converting enzyme inhibitors/Angiotensin receptor blockers		C09A, C09B, C09BB, C09BX, C09CA, C09DA, C09DB, C09DX, C09XA
Diuretics		C09BA, C09DA, C03A, C03B, C03C, C03D, C03E, C07B, C07C, C07D
Beta-blockers		C07A, C07B, C07C, C07D, C07E, C07F
Calcium channel blockers		C09BB, C09DB, C08C, C08D, C08E, C08G

© 2024 Hu JC et al. *JAMA Network Open.*

Lipid modifying agents	C10A, C10B
Oral antithrombotic agents	B01AA, B01AC, B01AE, B01AF, B01AX
Oral non-steroidal anti-inflammatory drugs	M01A
Systemic glucocorticoids	H02A, H02B
Proton pump inhibitors and Histamine 2-receptor blockers	A02BA, A02BC
Antiseizure medications	N03A
Methotrexate	L04AX03

Abbreviations: ATC: Anatomical Therapeutic Chemical

© 2024 Hu JC et al. *JAMA Network Open.*

eTable 7. ATC codes to identify exposure or comparator drugs

Drugs		ATC code
SGLT2 inhibitors	Empagliflozin	A10BK03, A10BD19, A10BD20, A10BD27
	Dapagliflozin	A10BK01, A10BD15, A10BD21, A10BD25
	Canagliflozin	A10BK02, A10BD16
	Ertugliflozin	A10BK04, A10BD23, A10BD24
GLP-1 RAs	Liraglutide	A10BJ02, A10AE56
	Lixisenatide	A10BJ03, A10AE54
	Dulaglutide	A10BJ05
	Semaglutide	A10BJ06

Abbreviations: ATC: Anatomical Therapeutic Chemical; GLP-1 RAs: Glucagon-like peptide-1 receptor agonists; SGLT2: Sodium-glucose cotransporter-2

© 2024 Hu JC et al. *JAMA Network Open.*

eTable 8. Composite anemia outcome definitions

Outcome	Components	Details
Anemia events	Laboratory data changes	Hemoglobin level < 12 g/dl for females, hemoglobin level < 13 g/dl for males
	Clinical diagnosis*	ICD-9-CM codes: 280-285<br>ICD-10-CM codes: D50-D64
Anemia treatments	Iron preparations	ATC codes: B03A
	Erythropoiesis-stimulating agents	ATC codes: B03XA
	Red blood cell transfusion	NHI codes: 93001C, 93002C, 93003C, 94001C

Abbreviations: ATC: Anatomical Therapeutic Chemical; ICD-9-CM: International Classification of Diseases, Ninth Revision, Clinical Modification; ICD-10-CM: International Classification of Diseases, Tenth Revision, Clinical Modification; NHI: National Health Insurance

Note: *Diagnoses recorded in the Chang Gung Research Database used ICD-9-CM codes prior to 2016, and ICD-10-CM codes thereafter.

© 2024 Hu JC et al. *JAMA Network Open.*

eTable 9. Individual effects of each SGLT2 inhibitor with regard to composite anemia outcomes

	Patients	Events	IR (95% CI) per 100 person-years	HR (95% CI)
**Empagliflozin**				
SGLT2 inhibitors	6,475	1,615	8.75 (8.33-9.19)	0.80 (0.72-0.89)
GLP-1 RAs	1,469	449	11.01 (10.01-12.07)	1 [Reference]
**Dapagliflozin**				
SGLT2 inhibitors	4,905	1,130	8.05 (7.59-8.54)	0.80 (0.72-0.90)
GLP-1 RAs	1,447	418	10.01 (9.07-11.02)	1 [Reference]
**Canagliflozin**				
SGLT2 inhibitors	842	187	9.69 (8.35-11.18)	0.76 (0.64-0.90)
GLP-1 RAs	1,416	468	11.99 (10.93-13.13)	1 [Reference]

Abbreviations: CI: Confidence interval; GLP-1 RAs: Glucagon-like peptide-1 receptor agonists; HR: Hazard ratio; IR: Incidence rate; SGLT2: Sodium-glucose cotransporter-2

© 2024 Hu JC et al. *JAMA Network Open.*

eTable 10. Sensitivity analysis of composite anemia outcomes

	Patients	Events	IR per 100 person-years (95% CI)	HR (95% CI)
**On-treatment analysis**				
SGLT2 inhibitors	12,331	1,868	6.87 (6.56-7.19)	0.66 (0.58-0.74)
GLP-1 RAs	1,468	286	10.93 (9.70-12.27)	1 [Reference]
**Excluding lixisenatide from the GLP-1 RA group**				
SGLT2 inhibitors	12,273	2,865	8.30 (8.00-8.61)	0.75 (0.68-0.83)
GLP-1 RAs	1,328	425	10.96 (9.94-12.06)	1 [Reference]
**Excluding remote anemia cases from 12 weeks to 1 year prior to the index date**				
SGLT2 inhibitors	11,180	2,231	6.85 (6.57-7.14)	0.84 (0.75-0.95)
GLP-1 RAs	1,287	313	8.09 (7.22-9.04)	1 [Reference]

Abbreviations: CI: Confidence interval; GLP-1 RAs: Glucagon-like peptide-1 receptor agonists; HR: Hazard ratio; IR: Incidence rate; SGLT2: Sodium-glucose cotransporter-2

© 2024 Hu JC et al. *JAMA Network Open.*
