# Supplementary material for: Use of SGLT2 Inhibitors vs GLP-1 RAs and Anemia in Patients With Diabetes and CKD
Source: JAMA Netw Open. 2024 Mar 4;7(3):e240946. doi: 10.1001/jamanetworkopen.2024.0946 (PMC10912959; doi:10.1001/jamanetworkopen.2024.0946)
Supplement: Supplement 2. — Data Sharing Statement [file jamanetwopen-e240946-s002.pdf]

Data Sharing Statement

Hu. Use of SGLT2 Inhibitors vs GLP-1 RAs and Anemia in Patients With Diabetes and CKD. *JAMA Netw Open*. Published March 04, 2024. doi:10.1001/jamanetworkopen.2024.0946

Data
Data available: No
